# Hierarchical neighbor effects on mycorrhizal community structure and function

**DOI:** 10.1002/ece3.2299

**Published:** 2016-07-05

**Authors:** Holly V. Moeller, Ian A. Dickie, Duane A. Peltzer, Tadashi Fukami

**Affiliations:** ^1^ Department of BiologyStanford UniversityStanfordCalifornia94305; ^2^ Bio‐Protection Research CentreLincoln UniversityLincoln7640New Zealand; ^3^ Landcare ResearchLincoln7640New Zealand; ^4^ EcologyEvolution & Marine BiologyUniversity of CaliforniaSanta BarbaraCalifornia93106; ^5^ Present address: Woods Hole Oceanographic Institution266 Woods Hole Road, Mail Stop 52Woods HoleMassachusetts02543

**Keywords:** Community assembly, community coalescence, community dynamics, compositional variation, ecosystem function, ectomycorrhizal fungi, plant–fungal interactions

## Abstract

Theory predicts that neighboring communities can shape one another's composition and function, for example, through the exchange of member species. However, empirical tests of the directionality and strength of these effects are rare. We determined the effects of neighboring communities on one another through experimental manipulation of a plant‐fungal model system. We first established distinct ectomycorrhizal fungal communities on Douglas‐fir seedlings that were initially grown in three soil environments. We then transplanted seedlings and mycorrhizal communities in a fully factorial experiment designed to quantify the direction and strength of neighbor effects by focusing on changes in fungal community species composition and implications for seedling growth (a proxy for community function). We found that neighbor effects on the composition and function of adjacent communities follow a dominance hierarchy. Specifically, mycorrhizal communities established from soils collected in Douglas‐fir plantations were both the least sensitive to neighbor effects, and exerted the strongest influence on their neighbors by driving convergence in neighbor community composition and increasing neighbor seedling vigor. These results demonstrate that asymmetric neighbor effects mediated by ecological history can determine both community composition and function.

## Introduction

By exchanging organisms, nutrients, and organic materials, neighboring communities can influence one another's species composition and function (Polis et al. 1997; Leibold et al. 2004). However, the principles governing the strength and directionality of these interactions remain relatively untested. Do these influences tend to be symmetric, such that equal exchange leads to equal influence between neighbors, or are they hierarchical, such that communities with certain species composition affect their neighbors more greatly than vice versa? For example, the compositional and functional outcomes of community coalescence (sensu Rillig et al. 2015) could be asymmetric in cases where communities follow a dominance hierarchy of influence strength that depends upon ecological history. Resolving this question would improve our understanding of community assembly and function (Rillig et al. 2015) by explaining the relative dominance or coexistence of neighboring communities. Moreover, experimental tests of neighbor effects are particularly informative for predicting outcomes at ecosystem and community boundaries, which are frequently impacted by both destructive (Haddad et al. 2015) and constructive (Barnes et al. 2014; Winsa et al. 2015) human influences.

Here, we present an empirical test for neighbor effects through experimental manipulation of mutualistic ectomycorrhizal (EM) fungal communities. In this experiment, intact tree seedling root systems were analogous to an “island” or “patch” with a bounded EM fungal community that is dynamic (Jones et al. 1997; Baar et al. 1999; Gehring et al. 2014) and affected by abiotic filters and interspecific competition (Kennedy 2010; Koide et al. 2011). These root system communities are known to interact belowground, including through the exchange of community members (Simard et al. 2012). Tree–fungal interactions may also be highly specific (Ishida et al. 2007; Tedersoo et al. 2008), leading to spatial heterogeneity in the EM fungal community that maps to heterogeneity in the tree canopy (Moeller et al. 2015; Pickles et al. 2015). Furthermore, partner quality varies among fungal taxa (Kennedy et al. 2007; Kipfer et al. 2012), so the composition of the fungal community has functional implications for the performance of the associated plant partner (Jonsson et al. 2001; Kranabetter et al. 2015) and, ultimately, for ecosystem processes such as primary productivity and nutrient cycling (Gehring et al. 2014).

**Figure 1:**
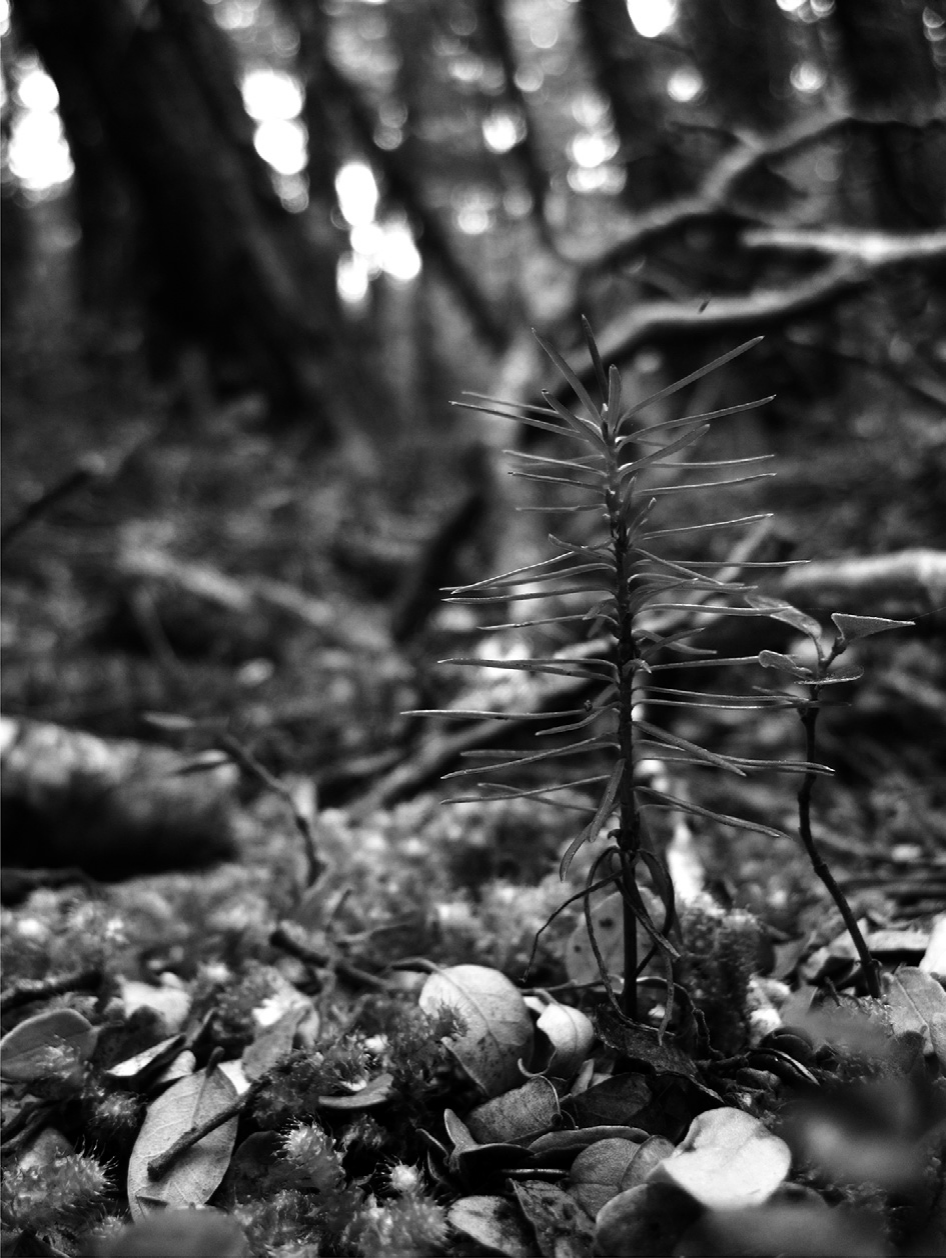
An invasive Douglas‐fir (*Pseudotsuga menziesii*) seedling establishing in a native southern Beech (*Fuscospora cliffortioides*) forest. Picture by H.V. Moeller, February 2012, Cora Lynn, New Zealand.

We took advantage of these features of the EM mutualism and a unique field setting on the South Island of New Zealand by working with Douglas‐fir (*Pseudotsuga menziesii*), an invasive North American tree (Froude 2011), and its associated EM fungi (Fig. 1). In a greenhouse experiment, we first grew Douglas‐fir seedlings in soils collected from three contrasting ecological contexts to establish different fungal communities and subsequently transplanted the seedlings and their associated fungi in pairs into neutral soils. We then studied the effects of the exchange of EM fungi between neighboring communities on fungal composition and plant performance. The initial fungal communities were established using soils collected from three contexts in New Zealand: (1) Douglas‐fir plantations, where trees and co‐introduced EM fungi have been established for decades (Chu‐Chou and Grace 1987); (2) native southern beech (*Fuscospora cliffortioides*) forests, which have a diverse, assembled complement of endemic fungi (McKenzie et al. 2000); and (3) grasslands, where the absence of EM host trees has prevented the assembly of an EM fungal community (Moeller et al. 2015).

The experiment was designed to test two hypotheses about neighbor effects. First, we hypothesized that neighbor effects would be hierarchical, with certain fungal communities exerting stronger influences on neighbors’ composition and function than others. We expected that these hierarchical neighbor effects would be a consequence of a competitive dominance hierarchy for root resources (sensu Dayton 1971) such that fungal communities from some types of neighbors consistently displaced species from other types. We expected that more dominant fungal communities would both themselves converge on a particular community composition and drive the directional convergence of their neighbors toward that same composition. Thus, dominant communities would influence both the mean (ultimate community composition) and variation (convergence on that composition) of their neighbors. In this study, we predicted that the fungal communities originating from Douglas‐fir plantations would be most dominant because they were initially established in soils most likely to have the greatest abundance of EM fungi capable of forming symbioses with Douglas‐fir (Nuñez et al. 2009; Dickie et al. 2010; Moeller et al. 2015). In addition, we expected these communities to be pre‐assembled: comprised of the most dominant, Douglas‐fir compatible, fungal taxa due to their relatively long (>20 year) plantation history (Huang et al. 2014) during which the most competitive fungi could displace other species. In contrast, we expected Grassland origin communities to be least dominant because the absence of EM hosts should have both limited the availability of EM inoculum and prevented the assembly of an EM community (Karst et al. 2014). We expected Beech origin communities to be of intermediate competitiveness because of the presence of an assembled native EM fungal community of which some members are known to be compatible with Douglas‐fir (Moeller et al. 2015).

Second, we hypothesized that dominant communities would enhance the symbiotic benefit to plants of neighboring communities. To test this hypothesis, we measured one type of community function: seedling performance (quantified using growth and foliar color). Seedling performance represents the outcome of the mutualistic interaction between the plant and its fungal partners, and has community and ecosystem implications as an indicator of seedling survival and primary production. This hypothesis is based on assuming a positive relationship between fungal competitive ability and fungal partner quality. Such an assumption runs counter to intuition about competition–function trade‐offs in mutualisms: Typically, “cheaters” – in this case, fungi that invest in their own growth and reproduction rather than in delivering nutrients and water to their tree partners – are thought to be more competitively dominant because they retain more resources to invest in competition and rapid growth (McGill 2005; Porter and Simms 2014). Indeed, the accumulation of low‐quality fungi on plant root systems can lead to negative feedbacks on conspecific host plants (Bever et al. 2001). In our experimental setting, however, we assumed a positive feedback based on preferential carbon allocation by host plants to high‐quality fungal partners (Kiers et al. 2011; Bever 2015), and the subsequent increase in abundance of these high‐quality partners. While monocultures, such as the Douglas‐fir plantations that we studied, can sometimes accumulate pathogens, in this case, we assumed that the relatively recent introduction of Douglas‐fir and co‐introduction of mutualistic fungi (Chu‐Chou and Grace 1987), together with the absence of Douglas‐fir compatible fungi in Beech and Grassland contexts, would enhance this positive feedback. Given this assumption, we expected that Douglas‐fir origin communities would consist of relatively high‐quality partners. Thus, directional transfer of high‐quality partners from these communities to neighbors would increase the performance of neighboring seedlings.

## Materials and Methods

**Figure 2:**
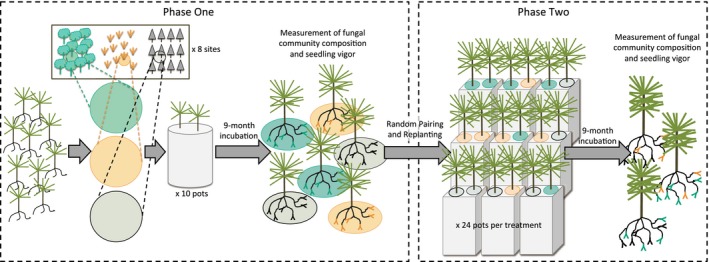
Diagram of experimental methods. During Phase 1 of the experiment, seedlings were planted in soil cores drawn from three different canopy types within eight sites. Seedlings grew and were colonized by fungi in the soil core for a period of 9 months. Seedlings were then removed from the soil cores and processed to (1) measure seedling performance as a proxy for community function and (2) determine the composition of the fungal community. During Phase 2 of the experiment, seedlings from Phase 1 were randomly paired and replanted in a common, sterile soil medium. Seedlings grew in this environment for 9 months, after which they were removed, functional measures were repeated, and fungal community composition was quantified.

To measure neighbor effects on composition and function, we first established different fungal communities on Douglas‐fir (*P. menziesii*) seedlings using field‐collected soils (Phase 1). We then randomly paired these fungal communities by replanting seedlings into sterile soil for a second greenhouse incubation period during which time the two fungal communities could interact (Phase 2). At the end of both phases, we quantified mycorrhizal fungal community composition by identifying fungi from root tips. We also quantified fungal community function by measuring seedling growth and foliar color. Because we worked in the introduced range of Douglas‐fir, we conducted our study in a contained greenhouse environment to avoid the ecological and ethical consequences of outplanting seedlings of a highly invasive species. Figure 2 provides a visual summary of the methods detailed below.

### Phase 1: Fungal community establishment

We collected intact soil cores from eight sites spanning a latitudinal gradient across the South Island of New Zealand in March 2012 (Fig. S1). At each site, we located the three ecological contexts within 1‐km of one another: (1) native southern beech (*F. cliffortioides*) forest; (2) established Douglas‐fir plantations; and (3) open grassland without a tree canopy. Within each context, we used random compass bearings and distances to locate and extract ten intact soil cores. Soil cores were extracted by hammering a 100‐mm length of 65‐mm‐diameter PVC pipe into the soil and then cutting the core free from below. Cores were immediately wrapped in plastic and, within 6 h, placed on ice for transport back to the laboratory, where they were stored at 4°C for up to 14 days. Handling equipment was bleach‐sterilized between core extractions.

When all 240 soil cores (8 sites × 3 contexts × 10 cores) had been collected from the field, a 1‐mm nylon mesh was secured to the bottom of each core, and each core was placed in its own individual foil tray in the greenhouse to avoid cross‐contamination. Twenty‐four controls (10 PVC pipe segments filled with autoclaved perlite and 14 filled with autoclaved soil homogenized from all 24 sampling locations) were added to the experiment at this time. Meanwhile, Douglas‐fir seeds (New Zealand Seed Trees, Rangiora, NZ, nzseeds.co.nz) were germinated in trays of autoclaved perlite. Ten days after germination, seedlings were transplanted into the soil cores in the greenhouse. Two seedlings were planted in each core, and transplant equipment was bleach‐sterilized between individual seedlings. After a 3‐week establishment period, any dead seedlings were replaced with new, live seedlings from a second round of seed germination so that two seedlings were maintained in each core. Seedlings were watered twice per week.

In December 2012 (9 months of growth; end of Phase 1), community composition and function were quantified. First, seedling height (mm) and needle‐bearing stem length (mm; the length of stem, including branches, on which needles were present) were measured as nondestructive proxies for seedling aboveground biomass. Later, destructive sampling revealed a strong relationship between these nondestructive proxies and traditional destructive metrics (*P *<* *0.001, *R* ^2^ = 0.698–0.864; Fig. S2). We also measured foliar color using a multinomial scale from 1 to 5, where 1 = yellowed and chlorotic, showing evidence of nutrient stress (Adams et al. 1999; Ashkannejhad and Horton 2006; Collier and Bidartondo 2009), and 5 = dark green needles (Fig. S3C).

Seedling pairs were then extracted from pots, and their root systems were gently separated and washed clean of soil using tap water. Any broken roots (i.e., roots that were no longer attached to a seedling and therefore could not be assigned to a host plant) were discarded. Each seedling was immediately processed under a dissection microscope. Root system length was determined by counting the number of root intersections with a 1‐cm grid (Newman 1966). Each root tip was examined and scored for the presence of a fungal hyphal mantle (which may indicate ectomycorrhization; we periodically verified this by inspection for a Hartig net using a compound microscope). Where mycorrhizas were present, up to 12 tips (but no more than half of the mycorrhizal root tips of a seedling) were randomly selected for DNA extraction based on proximity to randomly selected coordinates on the counting grid. This sampling approach was designed to capture the most abundant fungal taxa, while maximizing preservation of the fungal community for Phase 2.

### Phase 2: Fungal community mixing and replanting

During Phase 2, we set up six treatment groups representing all possible pairwise combinations of context origin (i.e., Beech–Beech, Beech–Douglas‐fir, Beech–Grassland, Douglas‐fir–Douglas‐fir, Douglas‐fir–Grassland, and Grassland–Grassland). At the end of Phase 1, we had sufficient survivorship to randomly assign 48 seedlings of each context origin to each of the three treatments containing that origin type. As a result, we had 24 pots each of Beech–Beech, Douglas‐fir–Douglas‐fir, and Grassland–Grassland treatments, and 48 pots each of Beech–Douglas‐fir, Beech–Grassland, and Douglas‐fir–Grassland treatments.

We minimized handling stress by replanting seedlings within 4 h of extraction from the Phase 1 soil core. Following the above measurements, seedlings were replanted into 1‐L pots filled with a 1:1 mix of autoclaved soil (collected from Craigieburn Forest, Fig. S1) and autoclaved sand. Following replanting, pots were topped with a 1‐cm layer of autoclaved perlite and placed in individual tin trays to avoid cross‐contamination between pots. We controlled for the soil bacterial community by inoculating the new pots with a 2 mL aliquot of homogenized flow‐through. This flow‐through was comprised of tap water that had passed through the Phase 1 pots just prior to harvest. Approximately equal amounts of flow‐through were collected from each of the 264 pots in the greenhouse (240 soil cores + 24 controls), filtered with a 2‐μm mesh to remove large eukaryotic soil biota and fungal spores, homogenized, and stored at 4°C during the harvest and replanting process.

Eight pots of paired control seedlings, which were initially grown in either sterilized perlite or sterilized soil during Phase 1, were processed identically to the other seedlings (i.e., harvested, washed, microscopically inspected, replanted in the sterile soil mixture, and inoculated with homogenized flow‐through). These seedlings were maintained during Phase 2 of the experiment to control for any fungal contamination in the greenhouse. In total, Phase 1 harvest and Phase 2 replanting were completed in 10 days.

Seedlings were incubated in the greenhouse in their Phase 2 pots for a further 9 months before the final harvest. At this time, nondestructive measures of seedling vigor described in Phase 1 were repeated. All root tips were examined for the presence of mycorrhizas. Twelve mycorrhizal root tips per mycorrhizal seedling were randomly selected for DNA extraction. Where fewer than twelve mycorrhizal root tips were present, all mycorrhizal tips were sampled. Seedling shoots and roots were separated at the root collar and dried (60°C, 72 h) to obtain dry biomass weight.

### Fungal identification

During microscope processing, each ectomycorrhizal root tip selected for identification was rinsed in tap water and placed directly into 10 μL Extraction Solution (SKU E7526; Sigma‐Aldrich Co. LLC, St. Louis, MO). We extracted DNA using extraction solution and neutralization solution B (Sigma‐Aldrich Co. LLC) following the protocol of Avis et al. (2003). The internal transcribed spacer (ITS) region of the nuclear ribosomal RNA genes of each root tip was amplified using the ITS‐1F (Gardes and Bruns 1993) and ITS‐4 primers (White et al. 1990), and sequenced by Beckman Coulter Genomics (Danvers, MA).

Sequences were clustered into operational taxonomic units (OTUs) using Geneious (Version 5.3.6; Biomatters, Auckland, New Zealand) at 97% sequence similarity. Because the 97% sequence similarity threshold has been shown to be a reasonable approximation for fungal species (Smith et al. 2007; Lekberg et al. 2014), we refer to these OTUs as species. Identity was assigned by comparing OTU sequences to the GenBank database (Benson et al. 2005) using the Basic Local Alignment And Search Tool (BLAST, http://blast.ncbi.nlm.nih.gov). We considered a match to be at species level if the percent homology was >97%; all sequences had >95% query coverage against the best match. We then screened our taxa to remove nonectomycorrhizal taxa based on Comandini et al. (2012).

### Data analysis

All statistical analyses were performed in R version 3.1.0 (R Core Team 2014). We used the package *vegan* (Oksanen et al. 2013) to compute compositional dissimilarity between all seedling‐hosted fungal communities (function *vegdist*) using Bray–Curtis, Jaccard, and Binomial indices; results were qualitatively similar among indices, so we report only results from analyses using the Bray–Curtis index.

To test hypothesis 1, we first used two‐dimensional nonmetric multidimensional scaling (2D‐NMDS) to visualize and quantify community composition and variability. We grouped seedlings by self × neighbor origin combination (giving nine seedling treatments: self = Beech × neighbor = Beech, self = Beech × neighbor = Douglas‐fir, self = Beech × neighbor = Grassland, etc.) and pooled seedlings by soil sampling site (Fig. S1) to evaluate shifts in community composition over time. We then quantified responses in both the mean and variation of fungal community composition. To test the hypothesis that the most dominant community would have a distinct community composition, and would make neighboring seedlings more similar to itself in fungal composition, we identified distinct compositional groupings in Phase 2 fungal communities using permutational multiple analysis of variance (PERMANOVA, function *adonis* in package *vegan*; Oksanen et al. 2013) followed by post hoc pairwise comparisons between treatment groups.

We tested for the order of the dominance hierarchy at three scales. First, we considered the entire experiment following Phase 2 and assessed the degree to which different treatment groups converged on a common community composition as an indication of which community was most dominant by reducing variation in its own and its neighbors’ community composition. We did this by first identifying the 2D‐NMDS centroid of all Phase 2 data, and then calculating bray distance (function *betadisper*) of each seedling from this centroid to determine the effects of treatment group on convergence on a common community composition. Second, we considered data within treatments (origin–origin combinations). We computed dispersion in species composition among fungal communities within‐treatment groups (function *betadisper*). Where, at the end of Phase 1, an uninfected seedling was partnered with an infected one, we assigned the pair the maximum Bray–Curtis dissimilarity score of 1. Third, we considered data within each pot by determining which of the paired seedlings was more influential in shaping the final (end of Phase 2) fungal community. We did this by comparing the fungal community of each seedling at the end of Phase 2 to itself at the end of Phase 1, and to its co‐planted seedling at the end of Phase 1. If, for example, the seedling's Phase 2 community resembled its own Phase 1 community more than its partner's Phase 1 community, then that seedling's community was considered to be more influenced by “self” than “partner.”

To test hypothesis 2, we used seedling growth and foliar color as proxies for seedling vigor. To measure mean effects, we used two‐way ANOVAs followed by post hoc Tukey's tests where significant effects were found. To achieve normality (assessed using Q–Q plots) and homoscedasticity (assessed using Levene's test), we log‐transformed length and biomass values before performing parametric statistical tests. We also tested for the importance of random effects (e.g., site effects from the 8 locations at which Phase 1 soils were collected) by comparing AIC values from *lme* and *gls* maximum‐likelihood models. Because inclusion of random effects did not change the significance of any model terms or reduce AIC values, these results are not reported. To measure functional variation, we used principal components analysis (package *bpca*, function *bpca*) to compress seedling stem height, needle‐bearing stem length, root system length, and foliar color into two axes. We then linked function to fungal community composition by comparing Euclidean distance in function to Bray–Curtis dissimilarity in fungal communities (using *betadisper* on distance matrices within‐treatment groups, and using a Mantel test on the whole community data set).

Finally, to understand fungal mechanisms underlying these compositional changes, we quantified changes in mycorrhization levels (i.e., proportion of root tips with mycorrhizas), the importance of key taxa to community composition (i.e., relationship between presence/absence and community distance), and competition/facilitation relationships between fungi (using Spearman's correlation). Analysis of fungal species population dynamics also served as a test distinguishing between the role of inoculum density (i.e., the number of mycorrhized root tips of each fungal species added to an experimental pot at the start of Phase 2) and competitive dominance among fungal taxa. Specifically, if fungal community dominance was a function of inoculum density, one would expect that at the end of Phase 2, the most dominant fungi would be the ones that had an initially higher mycorrhizal extent. That is, there would be a positive correlation between relative abundance at the start of Phase 2, and change in abundance over the course of Phase 2, and there would be no change in rank‐abundance order of species. Therefore, we calculated the abundance of each fungus as a proportion of root tips occupied within a pot, and compared these proportional abundances at the end of Phase 1 and at the end of Phase 2 using both linear models (function *lm*) and rank‐abundance diagrams for visualization.

## Results

During the study, 90 of 528 seedlings died; this mortality was independent of treatment group (Tukey's HSD, *P *>* *0.05). At the end of the experiment, 83% of noncontrol seedlings had ectomycorrhizas, with 52 ± 26 (mean ± SD) % of seedling root tips mycorrhizal when ectomycorrhizas were present. No ectomycorrhizal fungi were observed on control seedlings at any point during the study. Of 1883 root tips sequenced at the end of Phase 1, 1626 (87.4%) had usable sequences representing 79 fungal taxa, of which 37 species (comprising 90.8% of obtained sequences) were mycorrhizal (Figs. S4A, S5). During Phase 2, 3456 tips were sequenced, producing 3080 usable sequences (89.1%) representing 30 fungal taxa, of which 16 (comprising 98.0% of the obtained sequences) were mycorrhizal (Figs. S4B, S6). Among mycorrhizal seedlings, during Phase 1, mean per‐seedling species richness was 1.8, and during Phase 2, mean per‐seedling species richness was 1.6. Throughout the study, 95% of seedlings had a species richness between 1 and 4 fungal taxa.

### Hypothesis 1: Hierarchical effects on fungal composition

**Figure 3:**
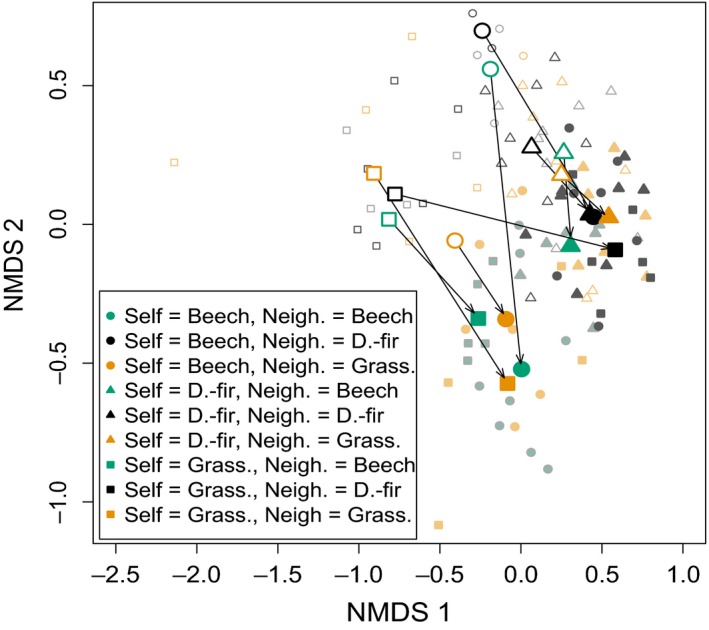
Nonmetric multidimensional scaling of fungal communities. Each small point represents a group of at least three seedlings originally planted in soils from a particular site that share a treatment group. For clarity, large points are placed at the centroid of each of the treatment groups. Hollow points represent fungal communities at the end of Phase 1, while filled points represent communities at the end of Phase 2. Arrows indicate shifts in community composition from Phase 1 to Phase 2 centroid, showing directional change and convergence in seedlings with Focal or Neighbor = Douglas‐fir origin.

**Figure 4:**
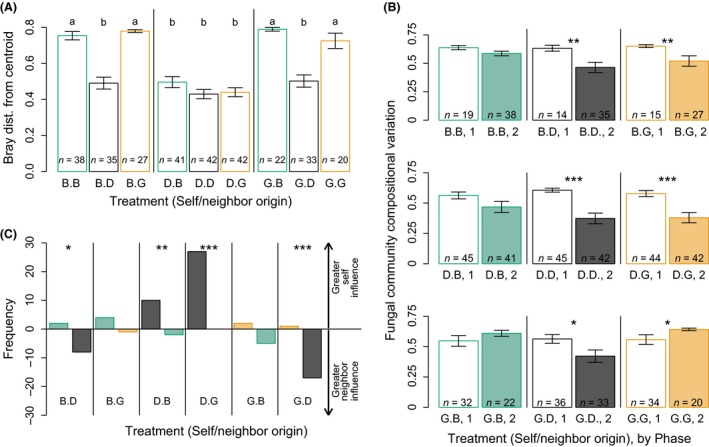
Treatment effects on fungal community composition. (A) Bray–Curtis distance from the overall experimental centroid, by treatment group. This measure of community dissimilarity is qualitatively similar to physical distance in Figure 3. Seedlings with Douglas‐fir influences show reduced distance. Letters indicate differences at the *P *<* *0.05 level (Tukey's HSD), and bar heights represent means, with whiskers indicating ±1 standard deviation. Numbers of seedlings are listed at the base of each bar. (B) Bar plots displaying within‐treatment fungal community compositional variation based on the Bray–Curtis dissimilarity index. Bar heights give means, with whiskers indicating ±1 standard deviation. Asterisks indicate significantly shifts in compositional variation from Phase 1 to Phase 2 (*t*‐test; *P <* *0.05; **P <* *0.01; ***P <* *0.001). Numbers of seedlings are listed at the base of each bar. (C) Evidence for a dominance hierarchy of neighbor influence. For each seedling, the relative influence of its own Phase 1 community versus its partner's Phase 1 community on its ultimate Phase 2 community composition was calculated by comparing Bray–Curtis dissimilarity. The dominant influence (self or partner) was determined based on the minimum dissimilarity value. Across treatments, seedling fungal communities were more likely to resemble the Phase 1 fungal community of the Douglas‐fir partner at the end of Phase 2. That is, Douglas‐fir origin fungal communities were most dominant. Asterisks indicate significantly uneven sample distributions (chi‐squared test, *P <* *0.05, **P <* *0.01, ***P <* *0.001).

From the end of Phase 1 to the end of Phase 2, fungal communities changed in ways that depended on both their identity and the identity of their neighbor (Fig. 3). In particular, Douglas‐fir origin communities (Fig. 3; triangles) and communities partnered with Douglas‐fir origin communities (Fig. 3; black symbols) converged in fungal community composition (Fig. 4A). This result was robust to sample size: when the dataset was randomly subsampled so that *n *=* *20 for all treatment groups, results were the same (data not shown).

Neighbor identity effects on composition were hierarchical. Within treatments, changes in compositional variation depended on neighbor identity. For example, variation among fungal communities of Beech origin seedlings paired with Douglas‐fir and Grassland origin seedlings decreased from the end of Phase 1 to the end of Phase 2, but variation among communities of Beech origin seedlings paired with other Beech origin seedlings did not (Fig. 4B). Similarly, variation among Douglas‐fir origin fungal communities decreased when paired with other Douglas‐fir origin communities and with Grassland origin communities, but not when paired with Beech origin communities (Fig. 4B). In contrast, while the variation among Grassland origin communities decreased when paired with Douglas‐fir origin communities, variation increased when paired with other Grassland origin communities (Fig. 4B). Within pot pairs, Douglas‐fir origin communities exerted the strongest influence on both their own and their neighbors’ fungal community composition, exhibiting consistent, directional transfer of fungal taxa to their neighbors during Phase 2 (Fig. 4C). In contrast, Grassland origin communities exerted the weakest neighbor effects, and were least likely to be the dominant influence on community composition (Fig. 4C).

Fungal community composition differed by treatment. Focal seedling origin was the only factor determining community composition following Phase 1, but both focal and neighbor origin identities, and their interaction, were significant following Phase 2 (Table 1). At the end of Phase 2, fungal communities of Douglas‐fir origin‐influenced seedlings (i.e., seedlings that were themselves Douglas‐fir origin, or had Douglas‐fir origin neighbors; Fig. 3 triangles or black symbols) had a significantly different composition than communities of seedlings without Douglas‐fir origin influences (PERMANOVA, composition ~ with or without Douglas‐fir origin influences, *P *<* *0.05). Seedlings in the Grassland–Grassland treatment group hosted distinct fungal communities from all other treatments (PERMANOVA, composition ~ Grassland–Grassland treatment group or other, *P *<* *0.05).

**Table 1:** Analysis of variance of fungal community composition. The best model was selected based on the significance of factors with a *P *<* *0.05 cutoff

Response variable	Best model	Factor	df	*F*‐statistic	*P*‐value	*R* ^2^
End of Phase 1 community composition^a^	Self	Self	2	11.287	<0.001	0.320
End of Phase 2 community composition^a^	Self × neighbor	Self	2	10.193	<0.001	0.204
		Neighbor	2	7.149	<0.001	0.143
		Self × neighbor	4	4.066	<0.001	0.163
Change in composition^b^	Self × neighbor	Self	2	15.917	<0.001	0.297
		Neighbor	2	4.184	0.003	0.078
		Self × neighbor	4	4.468	<0.001	0.167

a PERMANOVA analysis, using Bray–Curtis dissimilarity index.

b PERMANOVA analysis, using Euclidean distance metric.

### Hypothesis 2: Hierarchical effects on seedling performance

The identity of the focal seedlings was consistently the only predictor of community function proxies (indicators of seedling vigor: measures of seedling size and color) following Phase 1, except for seedling height, which was not well predicted by treatment (Table 2). Except for root system growth, function following Phase 2 and change in function (Phase 2 endpoint data – Phase 1 endpoint data) were best predicted by both focal seedling and neighbor seedling identity (Table 2).

**Table 2:** Analysis of variance of seedling vigor. Where necessary, data were log‐transformed to ensure normality and homoscedasticity before parametric statistical tests were applied. Model selection was based on the lowest AIC score and factor significance below the *P *<* *0.05 cutoff

Response variable	Transformation	Best model	*R* ^2^	Factor	df^a^	*F*‐statistic	*P*‐value
End of Phase 1							
Foliar color		Self	0.404	Self	2	121.400	<0.001
Needle‐bearing stem length (cm)	log	Self	0.235	Self	2	49.172	<0.001
Seedling height (cm)	log	–	–	–	–	–	–
Root system length (cm)	log	Self	0.198	Self	2	43.866	<0.001
End of Phase 2							
Foliar color		Self × neighbor	0.403	Self	2	88.671	<0.001
				Neighbor	2	13.435	<0.001
				Self × neighbor	4	8.374	<0.001
Dry root weight (g)	log	Self + neighbor	0.463	Self	2	147.522	<0.001
				Neighbor	2	5.251	0.006
Dry shoot weight (g)	log	Self+Neighbor	0.435	Self	2	132.200	<0.001
				Neighbor	2	3.648	0.027
Dry root:shoot ratio		Self + neighbor	0.365	Self	2	35.044	<0.001
				Neighbor	2	9.007	<0.001
Needle‐bearing stem length (cm)	log	Self × neighbor	0.409	Self	2	108.888	<0.001
				Neighbor	2	4.937	0.008
				Self × neighbor	4	2.650	0.033
Seedling height (cm)	log	Self × neighbor	0.375	Self	2	90.764	<0.001
				Neighbor	2	4.429	0.013
				Self × neighbor	4	4.457	0.002
Root system length (cm)	log	Self	0.398	Self	2	118.320	<0.001
Change over Phase 2							
Foliar color		Self × neighbor	0.109	Self	2	4.786	0.009
				Neighbor	2	7.868	<0.001
				Self × neighbor	4	4.392	0.002
Needle‐bearing stem length (cm)	log	Self + neighbor	0.298	Self	2	47.645	<0.001
				Neighbor	2	5.955	0.003
Root system size (cm)	log	Self	0.335	Self	2	90.152	<0.001
Seedling height (cm)	log	Self × neighbor	0.367	Self	2	57.420	<0.001
				Neighbor	2	8.110	<0.001
				Self × neighbor	4	3.346	0.011

Degrees of freedom. *N* = 360 for all analyses.

**Figure 5:**
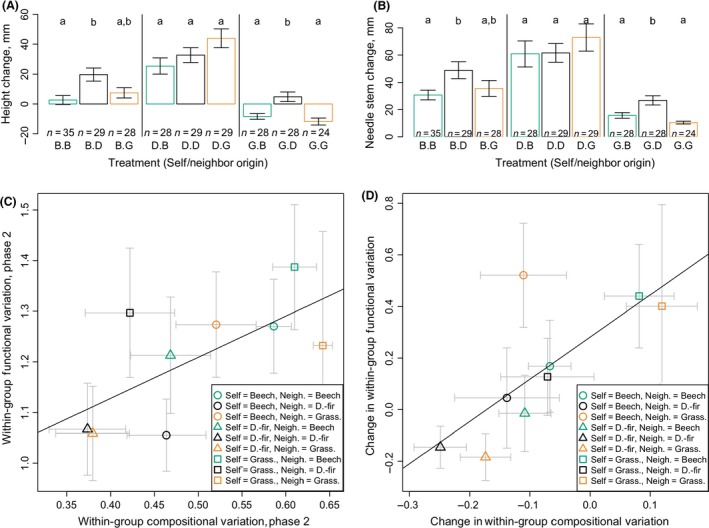
Community functional responses. From Phase 1 to Phase 2, (A) seedling height and (B) seedling needle‐bearing stem length increased most in the presence of Douglas‐fir influence (Self or Neighbor = Douglas‐fir origin). Bar heights give treatment means; whiskers indicate ±1 standard deviation. Letters indicate statistically significant differences in mean driven by neighbor effects (i.e., within focal origin groups, Tukey's HSD,* P* < 0.05). (C) At the end of Phase 2, variation in focal community function (beta dispersion of Euclidean distance based on foliar color, log[seedling height], log[seedling green stem length], and log[root system length]) increased with increasing within‐group compositional variation (*P* < 0.05, *R* ^2^ = 0.4556). (D) Change in compositional variation (Phase 2–Phase 1) and change in function variation (Phase 2–Phase 1) were also positively correlated (*P* < 0.05, *R* ^2^ = 0.5558).

Dominant neighbors (Douglas‐fir origin communities) increased the function of their partners, but were themselves relatively unaffected by neighbor influences. For example, Grassland origin seedlings were smaller than Beech and Douglas‐fir origin seedlings at the end of Phase 1 (Fig. S7A and C), but exhibited greater growth during Phase 2 when paired with Douglas‐fir origin seedlings, whereas Douglas‐fir origin seedlings were not influenced by neighbor identity (Fig. 5A and B). At the end of Phase 2, Douglas‐fir origin partners were also associated with improved foliar color across all focal seedling origins, whereas Grassland origin partners were associated with decreased foliar color in focal seedlings of Beech and Grassland origin (Fig. S7H).

Variation in seedling function (measured as Euclidean distance on a two‐dimensional PCA of performance, Fig. S8A) increased with increasing fungal community compositional variation. This was true across treatments (Fig. 5C), among all seedling pairs across the entire experiment (Fig. S8B), and within‐treatment groups (Fig. S9, Mantel simulated *P* < 0.001, based on 999 replicates). Change in within‐group compositional variation was also positively correlated with change in within‐group functional variation (Fig. 5D).

### Shifts in mycorrhization

**Figure 6:**
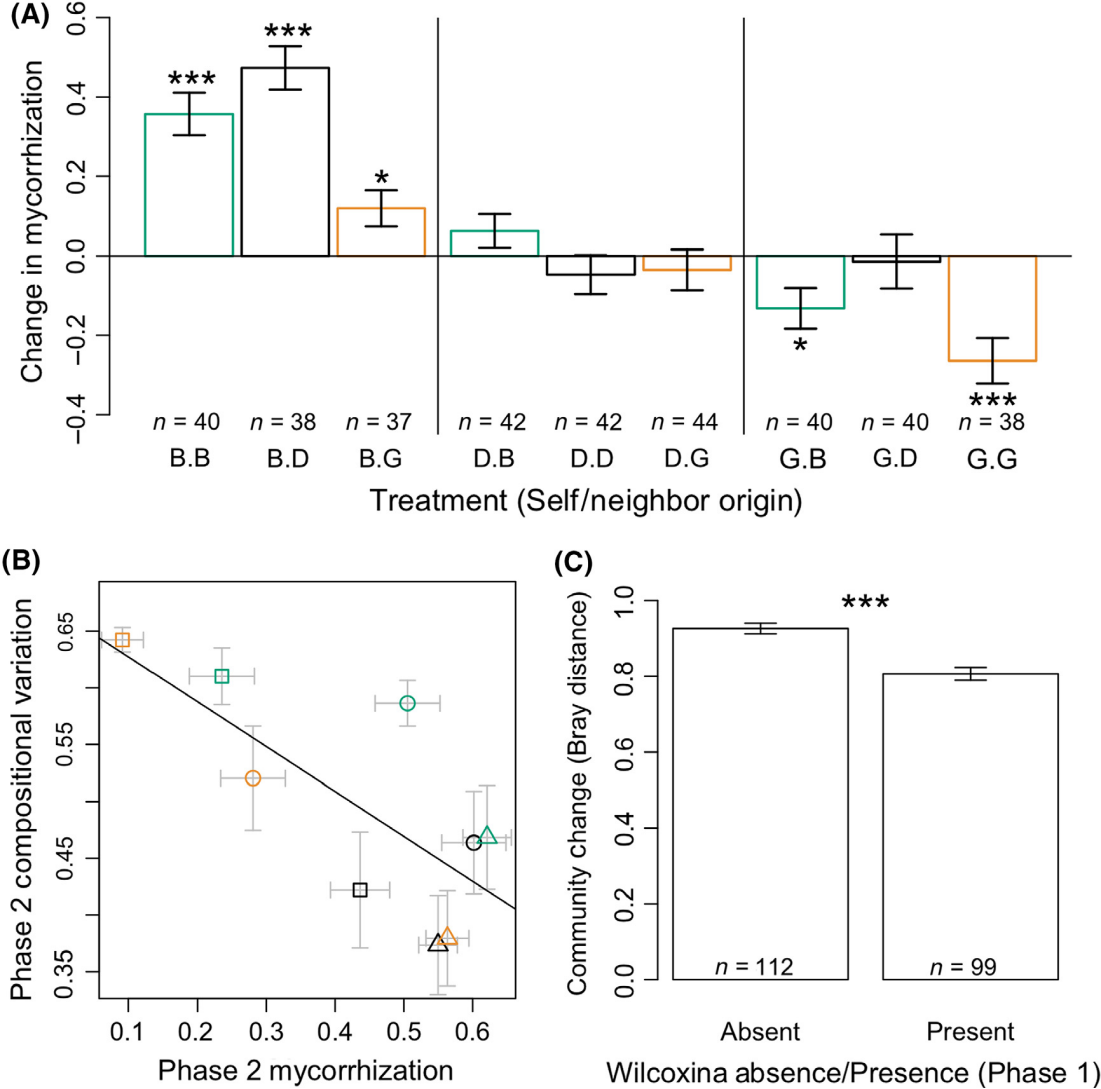
Fungal mycorrhization levels as potential mechanism for neighbor effects. (A) Change in mycorrhization levels (Phase 2–Phase 1) show that Douglas‐fir origin neighbors increase or stabilize mycorrhization levels compared to other neighbors. Bar heights are mean values with whiskers representing ±1 standard deviation. Asterisks indicate significant increases or decreases in mycorrhization (*t*‐test; *P <* *0.05; **P <* *0.01; ***P <* *0.001). (B) Within‐treatment groups at the end of Phase 2, mycorrhization was inversely correlated with compositional variation (*P *<* *0.05, *R* ^2^ = 0.4857). Symbols as in legend for Figure 5C and D. (C) The dominant fungal taxon, *Wilcoxina mikolae*, was associated with reduced shifts in the fungal community (Bray–Curtis dissimilarity between seedling community at the end of Phase 2 and end of Phase 1; *t*‐test statistic = 5.508, df = 198.4, *P *<* *0.001).

Total root system mycorrhization (proportion of seedling root tips colonized by any mycorrhizal fungal taxon) after Phase 1 was best predicted by seedling origin. However, mycorrhization after Phase 2 and change in mycorrhization over the course of Phase 2 were best predicted by models that accounted for self‐origin, neighbor origin, and their interaction (Table 3). Dominant neighbors increased mycorrhization of their pot partners (Fig. 6A). Generally, Beech origin seedling mycorrhization increased during Phase 2, but this increase was greatest for seedlings partnered with Douglas‐fir origin seedlings, and least for seedlings partnered with Grassland origin seedlings. Grassland origin seedling mycorrhization decreased during Phase 2, except when partners were of Douglas‐fir origin. Increased levels of mycorrhization were correlated with reduced compositional variation (Fig. 6B).

**Table 3:** Analysis of total root system mycorrhization (total proportion root tips infected by all fungi). Model selection was based on the lowest AIC score and factor significance below the *P *<* *0.05 cutoff

Response variable	Best model	*R* ^2^	Factor	df^a^	*F*‐statistic	*P*‐value
End of Phase 1 total mycorrhization (prop. root tips)	Self	0.292	Self	2	73.835	<0.001
End of Phase 2 total mycorrhization (prop. root tips)	Self × neighbor	0.335	Self	2	51.010	<0.001
			Neighbor	2	22.217	<0.001
			Self × neighbor	4	7.695	<0.001
Change in total mycorrhization over Phase 2	Self × neighbor	0.301	Self	2	57.145	<0.001
			Neighbor	2	11.253	<0.001
			Self × neighbor	4	3.684	0.006

a Degrees of freedom. *N* = 360 for all analyses.

Individual fungal taxa varied in their population dynamics over the course of Phase 2 (Fig. S10). Overall experimental diversity decreased (from 69 to 29 fungal taxa over the course of Phase 2; Fig. S4), and of the fungi that persisted through the end of Phase 2, interactions were predominantly negative (Fig. S11). Changes in abundance of each fungal taxon were best predicted by a combination of fungal species identity and inoculum density (measured as proportional abundance of mycorrhized root tips at the start of Phase 2), rather than inoculum density alone (Table 4). Indeed, the order of species when ranked by abundance changed over the course of Phase 2 for all but the Beech origin–Grassland origin treatment combination, providing further evidence that mechanisms besides initial abundance were involved in determining fungal community dominance (Fig. S12).

**Table 4:** Comparison of models for mycorrhization by species. Response variable is the change in mycorrhization by fungal taxon during Phase 2

Model	df^a^	*F*‐statistic	*P*‐value	*R* ^2^	AIC
Change in prop. inf. ~ Phase 1 prop. inf. by species	430	87.24	<0.001	0.167	140.143
Change in prop. inf. ~ Species identity	422	22.10	<0.001	0.306	69.138
Change in prop. inf. ~ Phase 1 prop. inf. by species × species identity	412	23.77	<0.001	0.501	−63.815

a Degrees of freedom. *N* = 432 for all analyses.

The most abundant fungal taxon in our study, *Wilcoxina mikolae*, was associated with decreased community compositional change (Fig. 6C) regardless of treatment (two‐way ANOVA showed nonsignificant focal or partner origin terms). That is, fungal communities that contained *W. mikolae* at the end of Phase 1 changed less over the course of Phase 2 than fungal communities that lacked *W. mikolae* at the end of Phase 1. In contrast, *Rhizopogon rogersii*, whose relative abundance declined during Phase 2 (Fig. S4), was associated with increased community change, although this association was only marginally significant (*P* = 0.06, Fig. S13).

## Discussion

We demonstrate empirically that neighboring communities can shape one another's composition and function through the exchange of member species. Further, we demonstrate that the compositional and functional outcomes of this community coalescence (sensu Rillig et al. 2015) can depend upon ecological history in predictable ways. That these effects follow a dominance hierarchy, hypothesized based on the ecological history of our study contexts, suggests that it may be possible to identify, a priori, systems in which asymmetric neighbor effects increase predictability. Both of our hypotheses were supported. First, we found that transfer of fungal species between communities was directional, following a hierarchy in which Douglas‐fir origin communities were most dominant, and Grassland origin communities were least dominant. Second, we found that dominant communities more strongly affected the function of neighboring communities. These findings shed new light on compositional and functional outcomes of community coalescence as outlined below.

The order of the community hierarchy fit our expectation based upon assembly history (e.g., Peay et al. 2011) that Douglas‐fir plantation communities would both dominate and most strongly shape fungal communities compared to native forest or grassland communities. Because the fungi present in our study were spore‐based colonizers predominantly associated with early‐successional stages (Moeller et al. 2015), persistence of this hierarchy may be a result of differential Douglas‐fir invasion history across ecological contexts. The Douglas‐fir plantations, whose soils produced the most dominant communities in our study, contain a spore bank that has been established over many years and includes a larger complement of mutualistic partners than other contexts, particularly grasslands, which are likely relatively spore‐depauperate (Collier and Bidartondo 2009; Nuñez et al. 2009; Huang et al. 2014; Karst et al. 2014). For example, the most abundant fungus in our study, *W. mikolae*, which is tolerant of ecosystem disturbance (Barker et al. 2012), has been widely co‐introduced to Douglas‐fir plantations throughout the southern hemisphere (Nuñez et al. 2009), including in New Zealand (Walbert et al. 2010; Moeller et al. 2015). Contrary to our expectations, native Beech‐associated fungi did not contribute to that community's intermediate position in the dominance hierarchy. Although some Beech‐associated fungi are able to form ectomycorrhizal associations with Douglas‐fir in the field (Moeller et al. 2015), in our study, the absence of these fungi may have been due to the absence of neighboring adult trees which may provide a carbon source necessary for establishment (Moeller et al. 2015), and/or incompatibility of these native fungi with greenhouse or experimental conditions. However, Beech origin communities may still have been of intermediate competitiveness in our study because of local establishment of introduced fungi on native Beech roots (Johnston 2009) and subsequent development of the inoculum bank (Huang et al. 2014). Grassland origin communities, while able to establish during Phase 1 of our study, declined in species richness and abundance unless paired with Beech or Douglas‐fir origin neighbors, suggesting that their initial membership was comprised of fungi that did not tolerate greenhouse conditions.

Dominant fungal communities decreased compositional variation among their neighbors by driving directional convergence of communities to a common composition. Our ability to detect these differences was likely enhanced by the relatively low diversity of the fungal community, which comprised 16 ectomycorrhizal taxa by the experiment's end, compared to 78 taxa associating with invasive Douglas‐fir seedlings in situ (Moeller et al. 2015). While this in situ diversity is low compared to the diversity observed in Douglas‐fir's native range, where studies of similar sampling intensity have identified >250 fungi by sporocarp alone (Smith et al. 2002), our bioassay total species richness is similar to that observed in seedling outplanting studies conducted in Douglas‐fir's native range. For example, Simard et al. (1997) identified 17 ectomycorrhizal morphotypes, and Pilz and Perry (1984) identified twelve. The simplification we observed over the course of our experiment may have been driven by competitive interactions among fungi, differences in fungal tolerance of the greenhouse environment, selectivity of mycorrhizal partners by the seedling hosts, or loss of rare taxa because of Phase 1 sampling. Specifically, our fungal identification method required the destructive sampling of seedling root tips, which would have reduced root tip inoculum density, particularly on seedlings where Phase 1 mycorrhization was already low. This could also have contributed to observed patterns in community dominance, with more highly mycorrhized Douglas‐fir origin seedling root systems (Moeller et al. 2015) having greater inoculum density in Phase 2.

Our observed reduction in fungal species richness differs from field observations that mycorrhizal fungal diversity on seedlings increases with seedling age (Moeller et al. 2015), and that forests accumulate fungal diversity as they age (Huang et al. 2014). There are at least two explanations for this difference. First, our closed greenhouse system lacked an external source of fungal propagules, which continually arrive in natural systems, and this may have limited the potential pool of fungal partners. This effect is particularly significant because experimental conditions favored spore‐based colonists (Moeller et al. 2015), eliminating many late‐successional species that tend to colonize by hyphal spread from adult host plants that serve as a carbon source (Simard et al. 1997; Taylor and Bruns 1999). Second, the timescale of our study was relatively short and included equivalently aged seedlings that, initially, had small root systems, forcing fungi into close physical proximity and resource competition. As a consequence, per‐seedling community diversity (species richness, or *α*‐diversity) was relatively low throughout the study, and there may have been little opportunity for less competitive fungi to persist. In addition, because our approach used a sampling methodology (root tip selection) targeted toward active ectomycorrhizal fungi, our dataset does not provide information on fungal taxa that may have been present in the soil as spores or that did not form mycorrhized root tips.

Consistent with our second hypothesis, dominant fungal communities increased neighboring seedling performance while reducing variation in performance. Because fungal identity and seedling growth are coupled by resource exchange, this was likely a consequence of the dominant neighboring community driving the convergence of fungal community composition. In our study, the dominant fungal taxa found within Douglas‐fir origin communities were associated with greater seedling performance, as well as with greater neighbor performance linked to the directional transfer of these fungi to neighboring communities. This positive association may have been the result of inherent fungal traits (i.e., more competitive fungi were also higher quality partners; see, e.g., Kennedy et al. 2007) or the result of preferential carbon allocation by the host seedlings to higher quality partners (as has been demonstrated in arbuscular mycorrhizae; see, e.g., Kiers et al. 2011; Fellbaum et al. 2014), enhancing the competitiveness of those fungi. In either case, increasing seedling performance may have served as a positive feedback to the coupled belowground community, as larger seedlings should provide more photosynthate belowground than smaller seedlings. This feedback could also have extended directly to neighbors: once a common mycorrhizal network was established between seedlings, larger, more vigorous Douglas‐fir origin seedlings could have supplied carbon to both fungal and seedling neighbors (Simard et al. 2012; Wu et al. 2012).

Our study is not the first to demonstrate neighbor effects on community composition or function. Particularly in mycorrhizal systems, where belowground mycorrhizal networks allow for the exchange of propagules and resources, neighbors are known to enhance seedling mycorrhization and growth (Horton et al. 1999; Simard et al. 2012) both through the direct supply of carbon and through the sharing of other nutrients such as nitrogen and phosphorus (Newman 1988; Fellbaum et al. 2014). However, previous studies of propagule exchange typically focus on clearly unidirectional effects (e.g., from mycorrhized to unmycorrhized root systems) of adult trees on seedlings. In contrast, our experiment evaluated neighbor effects across same‐age communities (i.e., neighbors with equal amounts of ecological history). Because of this design, our results demonstrate that neighbor effects on community structure and function can be predictably hierarchical.

## Conflict of Interest

None declared.

## Supporting information


**Figure S1.** Map of sampling sites on the South Island of New Zealand.
**Figure S2.** Correlation between non‐destructive and destructive seedling performance measures.
**Figure S3.** Seedling sampling methodology.
**Figure S4.** Rank‐abundance diagrams for fungal communities at the end of Phase 1 (top) and the end of Phase 2 (bottom).
**Figure S5.** Boxplots showing the abundance, measured as root tips mycorrhized by that fungus per seedling, of each of the 11 most abundant fungal taxa at the end of Phase 1.
**Figure S6.** Boxplots showing the abundance, measured as root tips mycorrhized by that fungus per seedling, of each of the 11 most abundant fungal taxa at the end of Phase 2.
**Figure S7.** Seedling performance responses during Phase 1 (panels A–D) and Phase 2 (panels E–K).
**Figure S8.** Seedling performance responses and variation at the across‐treatment scale.
**Figure S9.** Mantel tests across the study and partitioned by treatment for phase one (top 10 panels) and phase two (bottom 10 panels).
**Figure S10.** Changes in fungal populations on individual seedling root systems.
**Figure S11.** Correlogram showing primarily negative relationships between fungal taxa in the study.
**Figure S12.** Proportion of root tips in each pot occupied by each of the ten most abundant fungal taxa in the study.
**Figure S13.** *Rhizopogon rogersii* effect on change in community composition.
