# Supplementary material for: Hierarchical neighbor effects on mycorrhizal community structure and function
Source: Ecol Evol. 2016 Jul 5;6(15):5416–30. doi: 10.1002/ece3.2299 (PMC4984514; doi:10.1002/ece3.2299)
Supplement: Supplementary file 1 — Figure S1. Map of sampling sites on the South Island of New Zealand. Figure S2. Correlation between non‐destructive and destructive seedling performance measures. Figure S3. Seedling sampling methodology. Figure S4. Rank‐abundance diagrams for fungal communities at the end of Phase 1 (top) and the end of Phase 2 (bottom). Figure S5. Boxplots showing the abundance, measured as root tips mycorrhized by that fungus per seedling, of each of the 11 most abundant fungal taxa at the end of Phase 1. Figure S6. Boxplots showing the abundance, measured as root tips mycorrhized by that fungus per seedling, of each of the 11 most abundant fungal taxa at the end of Phase 2. Figure S7. Seedling performance responses during Phase 1 (panels A–D) and Phase 2 (panels E–K). Figure S8. Seedling performance responses and variation at the across‐treatment scale. Figure S9. Mantel tests across the study and partitioned by treatment for phase one (top 10 panels) and phase two (bottom 10 panels). Figure S10. Changes in fungal populations on individual seedling root systems. Figure S11. Correlogram showing primarily negative relationships between fungal taxa in the study. Figure S12. Proportion of root tips in each pot occupied by each of the ten most abundant fungal taxa in the study. Figure S13. Rhizopogon rogersii effect on change in community composition. [file ECE3-6-5416-s001.pdf]

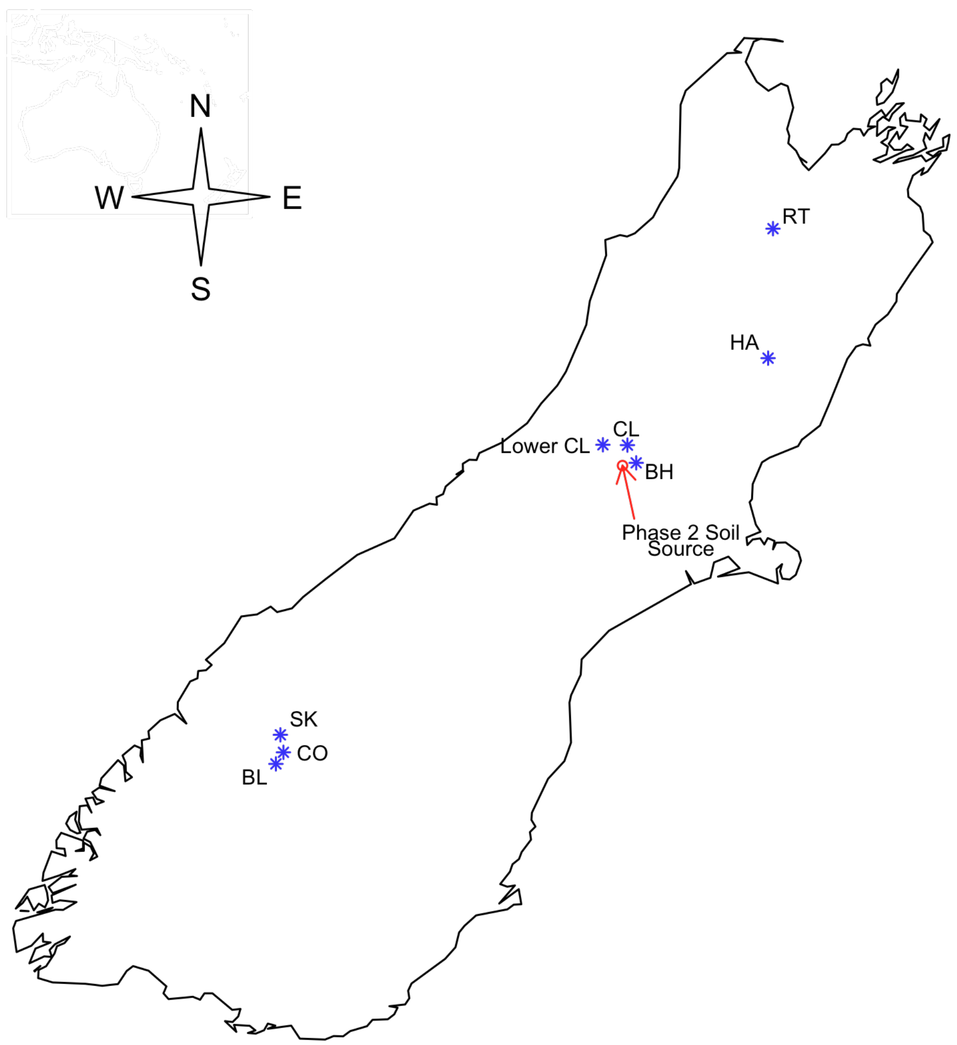

Supplemental Figure 1. Map of sampling sites on the South Island of New Zealand. Note the latitudinal stratification of sites. RT = Route 63, HA = Hanmer Springs, CL = Cora Lynn, BH = Bridge Hill, SK = Skippers Canyon, CO = Coronet, BL = Ben Lomond. The location for harvest of Phase 2 common soil is indicated by a red circle.

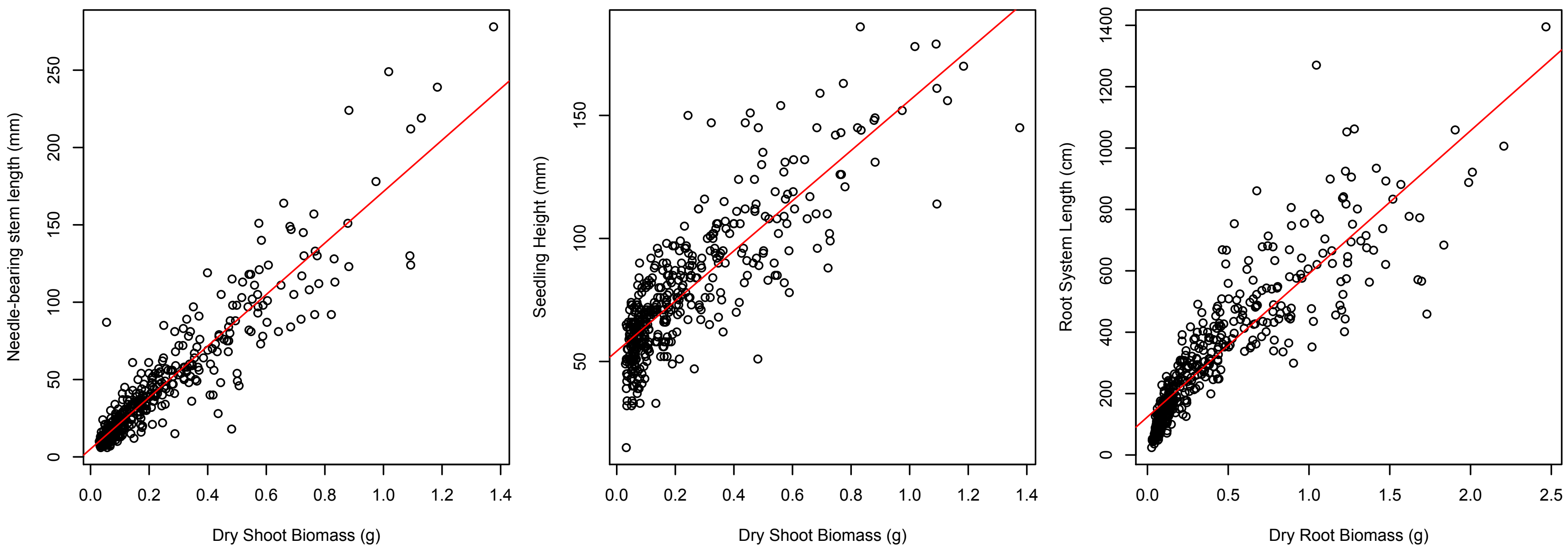

Supplemental Figure 2. Correlation between non-destructive and destructive seedling performance measures. For all panels, *P* << 0.001. R^2 values are 0.8639, 0.6979, and 0.7848 for panels **a-c**, respectively.

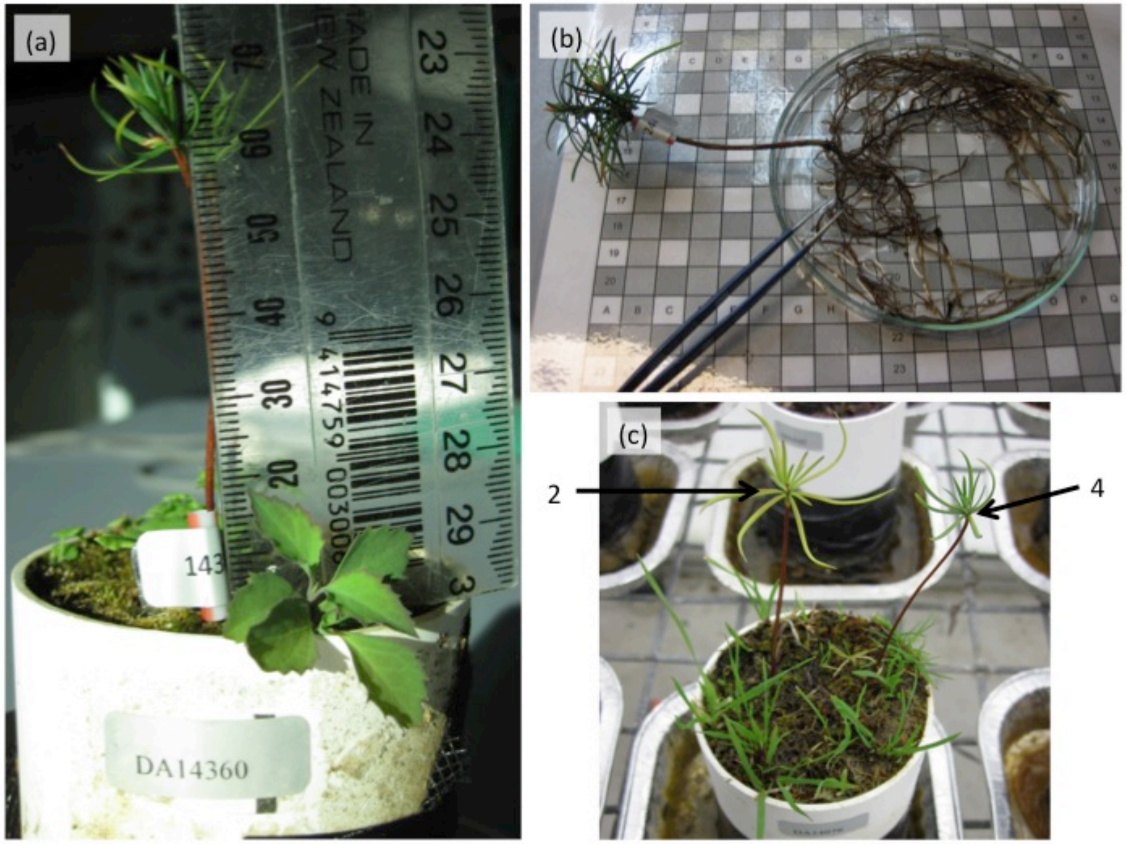

Supplemental Figure 3. Seedling sampling methodology. **(a)** Non-destructive measures of seedling performance included a measure of total seedling height (from pot soil surface to the tip of the most upright bud), and needle-bearing stem length (from the base of needles to the top of the bud tip, and lengths of any needle-bearing branches). **(b)** Seedling root systems were washed clear of soil and examined under a dissecting microscope. Intersections with a 1-cm grid were counted to determine root system length. The grid was also used as a coordinate system for random sampling of root tips when fungal colonization (i.e., a hyphal mantle) was observed. **(c)** Foliar color was scored based on seedling greenness on a scale from 0 to 5. 0 indicated a dead seedling (brown, brittle needles); live seedling scores ranged from 1 (extremely chlorotic) to 5 (dark green). Examples of seedlings scoring a 2 (left) and 4 (right) are shown.

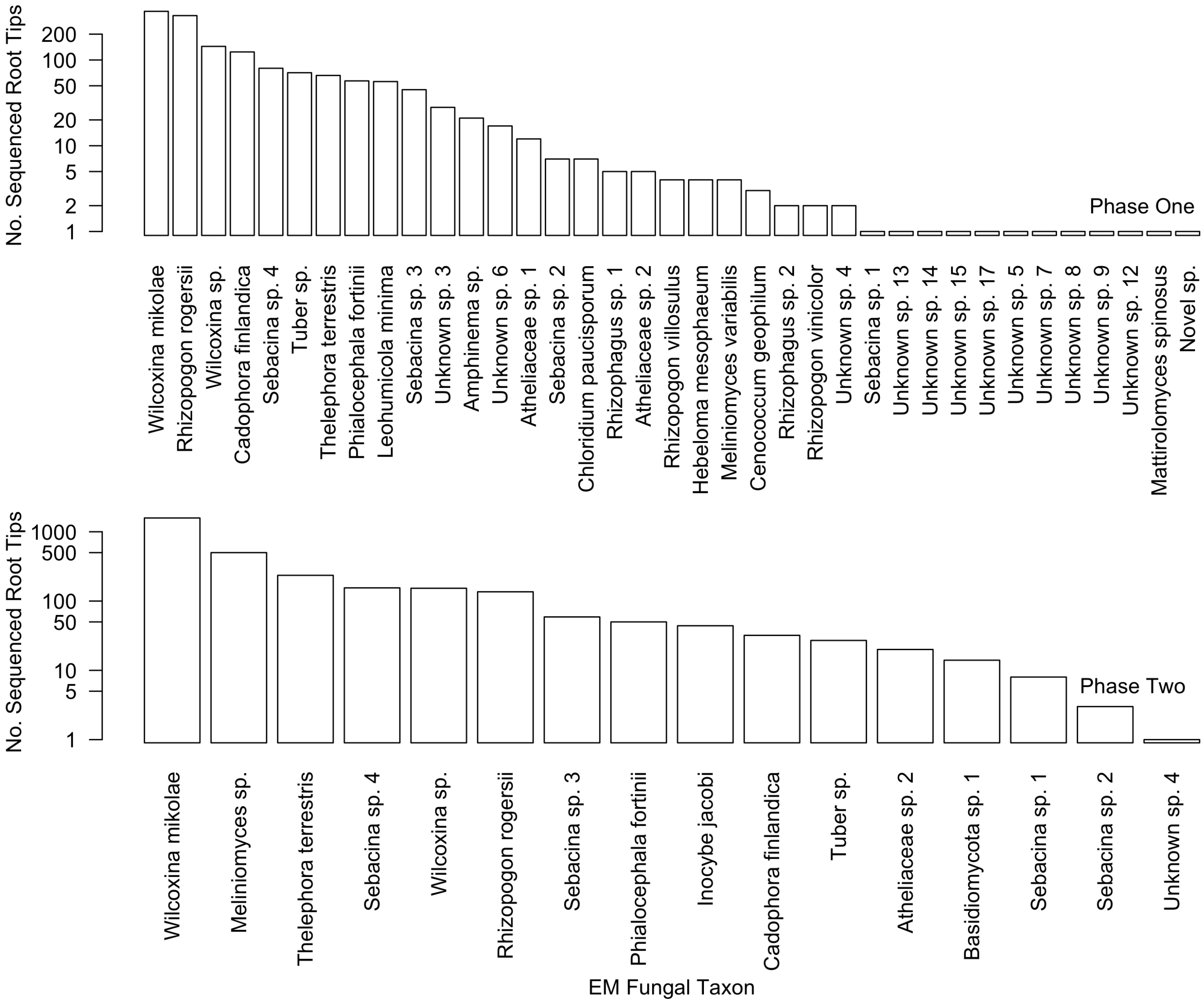

Supplemental Figure 4. Rank-abundance diagrams for fungal communities at the end of Phase 1 (top) and the end of Phase 2 (bottom).

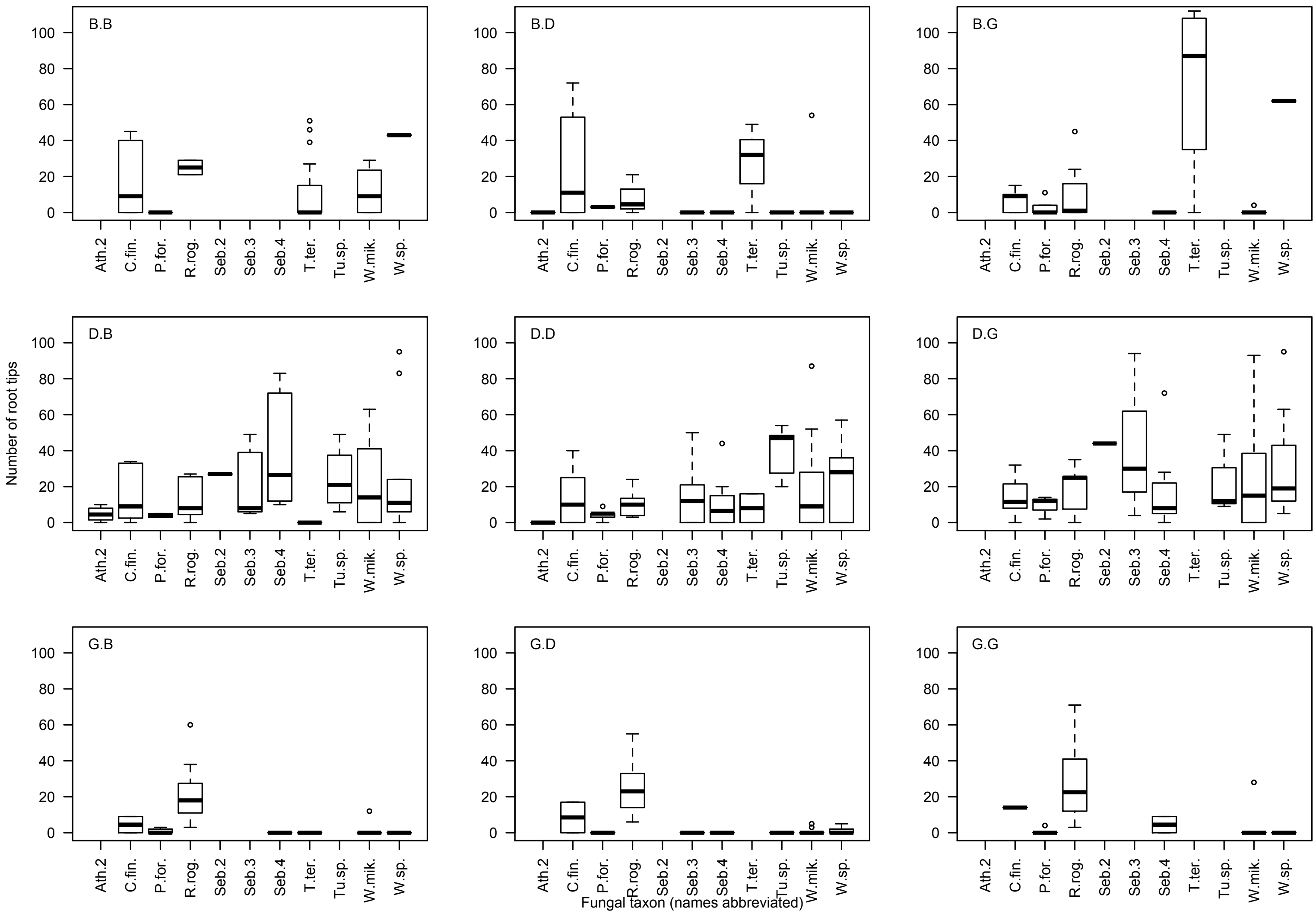

Supplemental Figure 5. Boxplots showing the abundance, measured as root tips mycorrhized by that fungus per seedling, of each of the 11 most abundant fungal taxa at the end of Phase 1. Data are partitioned by treatment (Self Origin . Neighbor Origin, as labeled in the upper left corner of each panel). Boxes are centered at median abundance values and extend to the first and third quartiles. Whiskers extend to minima and maxima, excluding outliers, which are plotted as points.

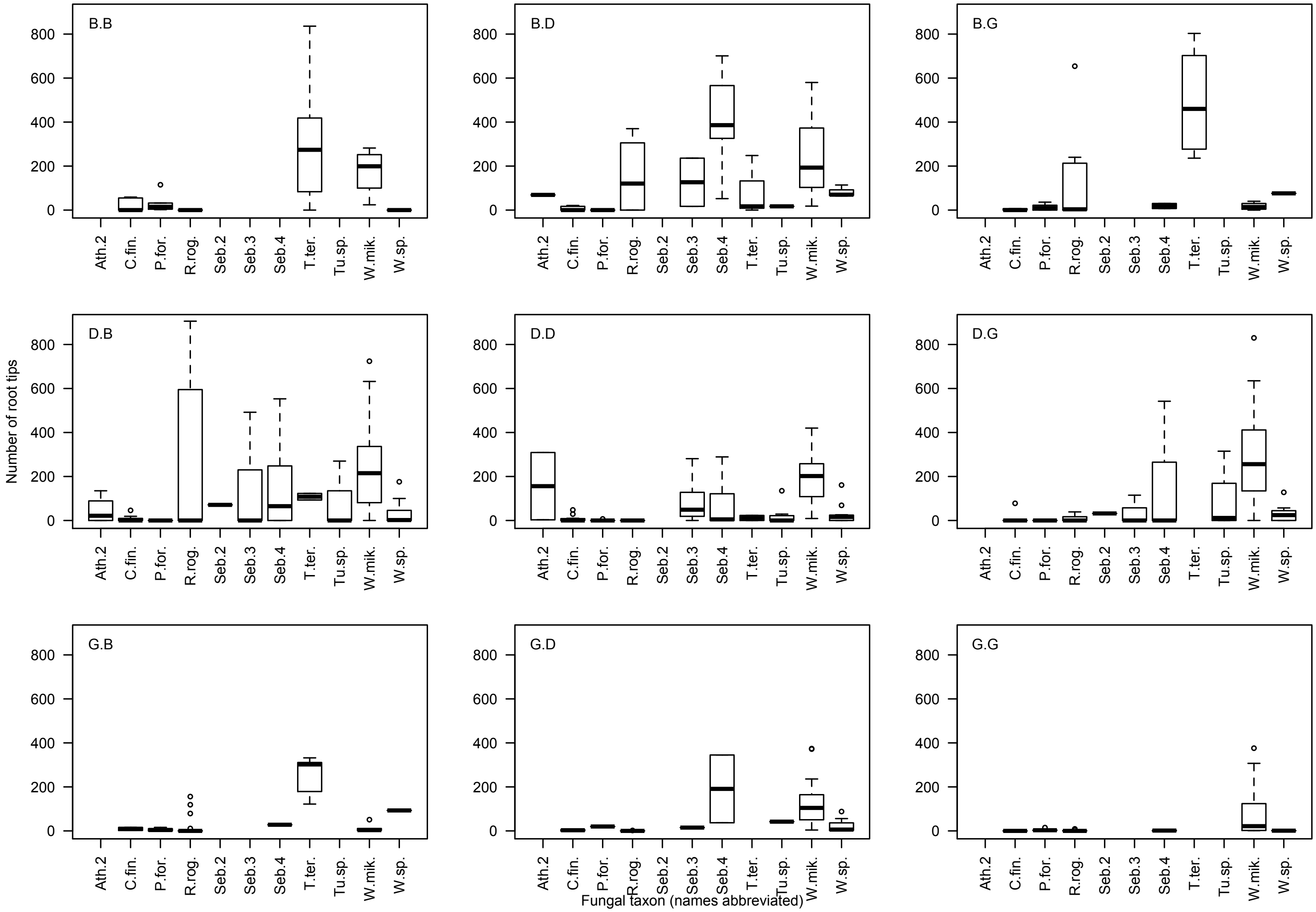

Supplemental Figure 6. Boxplots showing the abundance, measured as root tips mycorrhized by that fungus per seedling, of each of the 11 most abundant fungal taxa at the end of Phase 2. Data are partitioned by treatment (Self Origin . Neighbor Origin, as labeled in the upper left corner of each panel). Boxes are centered at median abundance values and extend to the first and third quartiles. Whiskers extend to minima and maxima, excluding outliers, which are plotted as points.

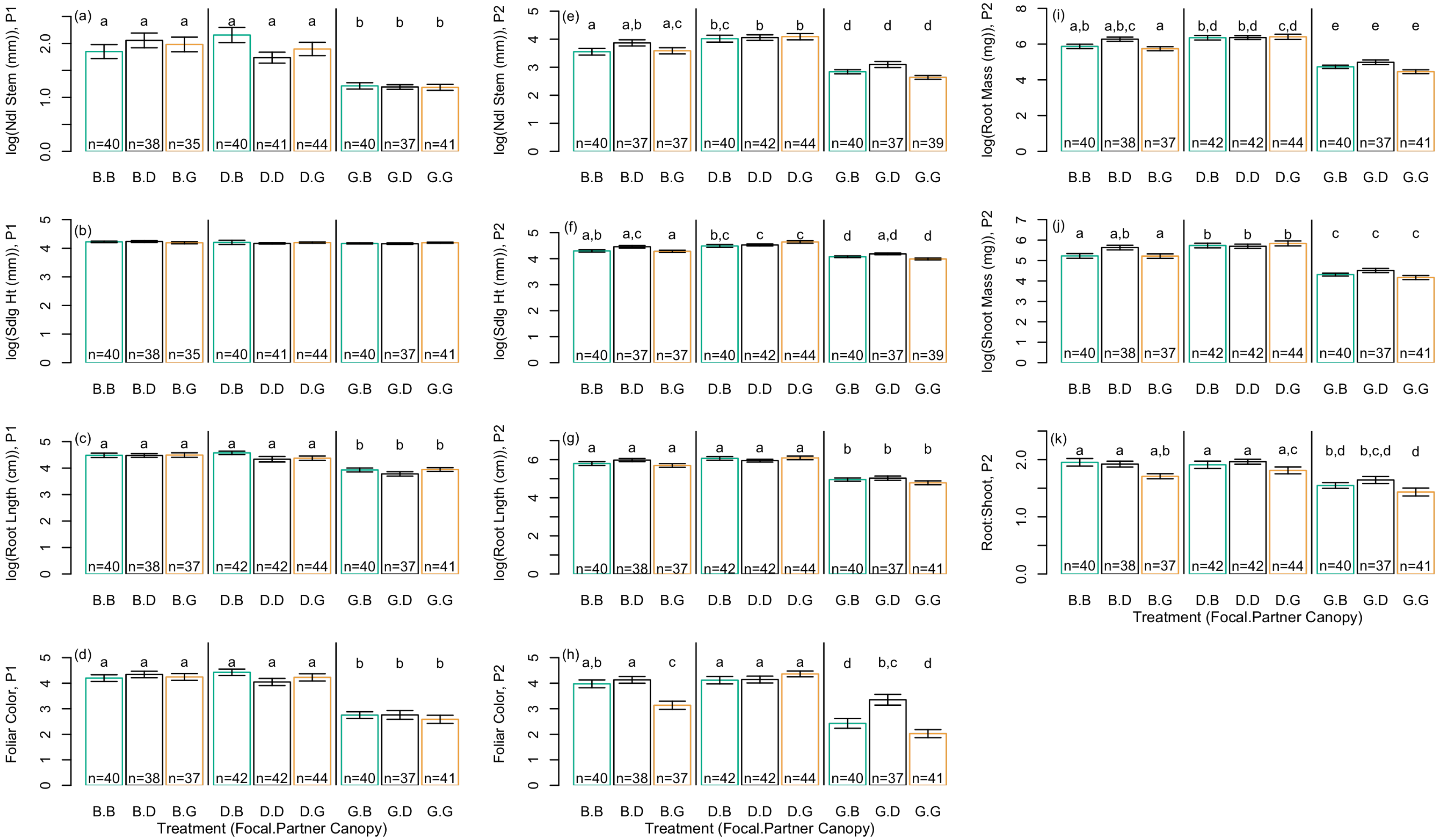

Supplemental Figure 7. Seedling performance responses during Phase 1 (panels a-d) and Phase 2 (panels e-k). Note that at the end of Phase 2, destructive harvest of seedlings allowed for measurements of dry biomass (panels i-k). Bar heights are mean values with whiskers representing ± 1 standard deviation. Numbers of seedlings in each treatment group are listed at the base of each bar. Letters indicate significant differences at the $P < 0.05$ level (Tukey's HSD).

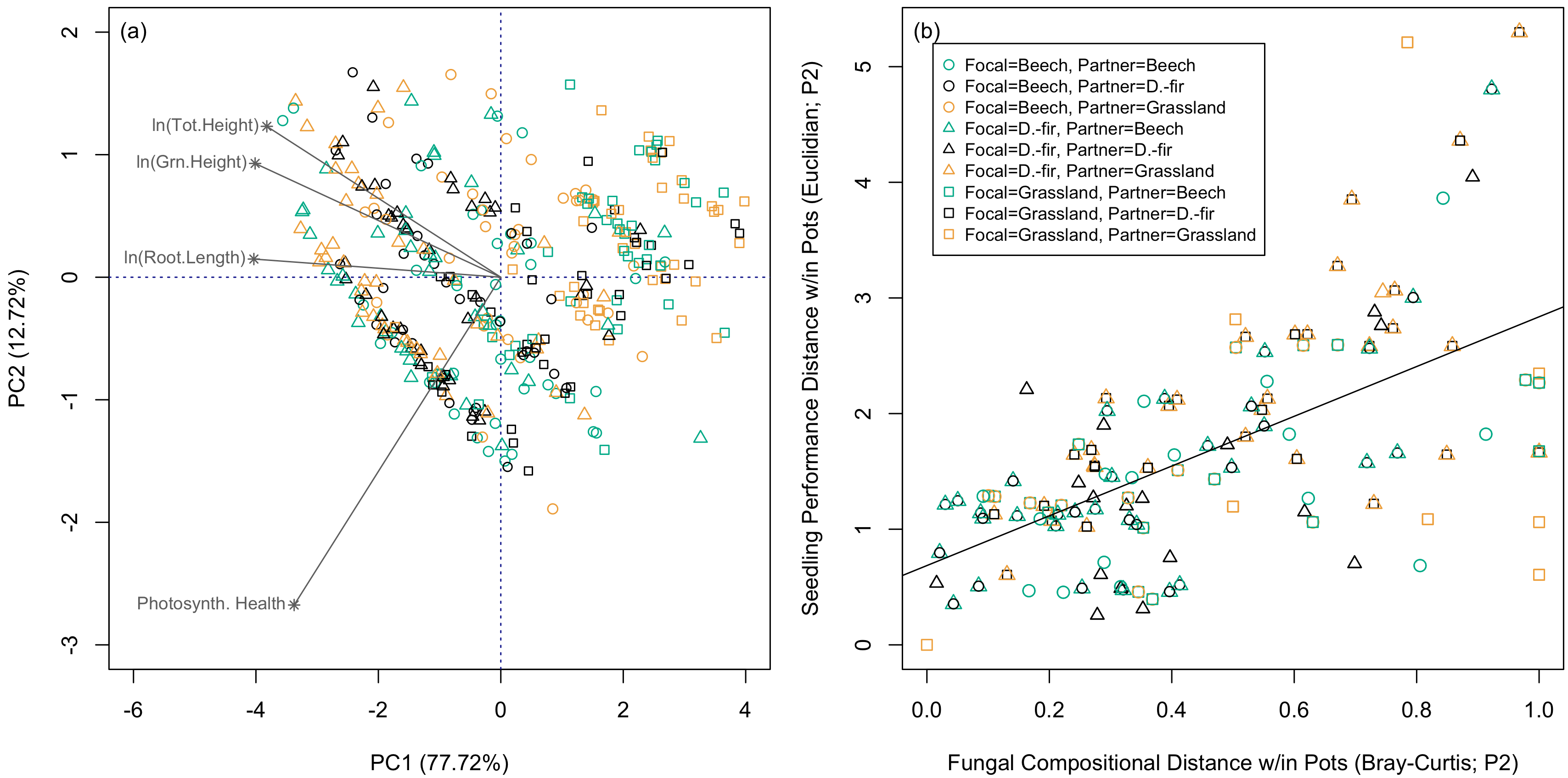

Supplemental Figure 8. Seedling performance responses and variation at the across-treatment scale. **(a)** Principal components analysis based on four non-destructive performance measures shows increased performance of seedlings with Douglas-fir ecological histories compared to seedlings that experienced Grassland conditions during Phase 1. **(b)** Linear relationship between distance in fungal communities (x-axis; Bray-Curtis dissimilarity) and distance in performance (y-axis; Euclidian distance) within pots (i.e., between seedling pairs) ($P < 0.001$, $R^2 = 0.3404$).

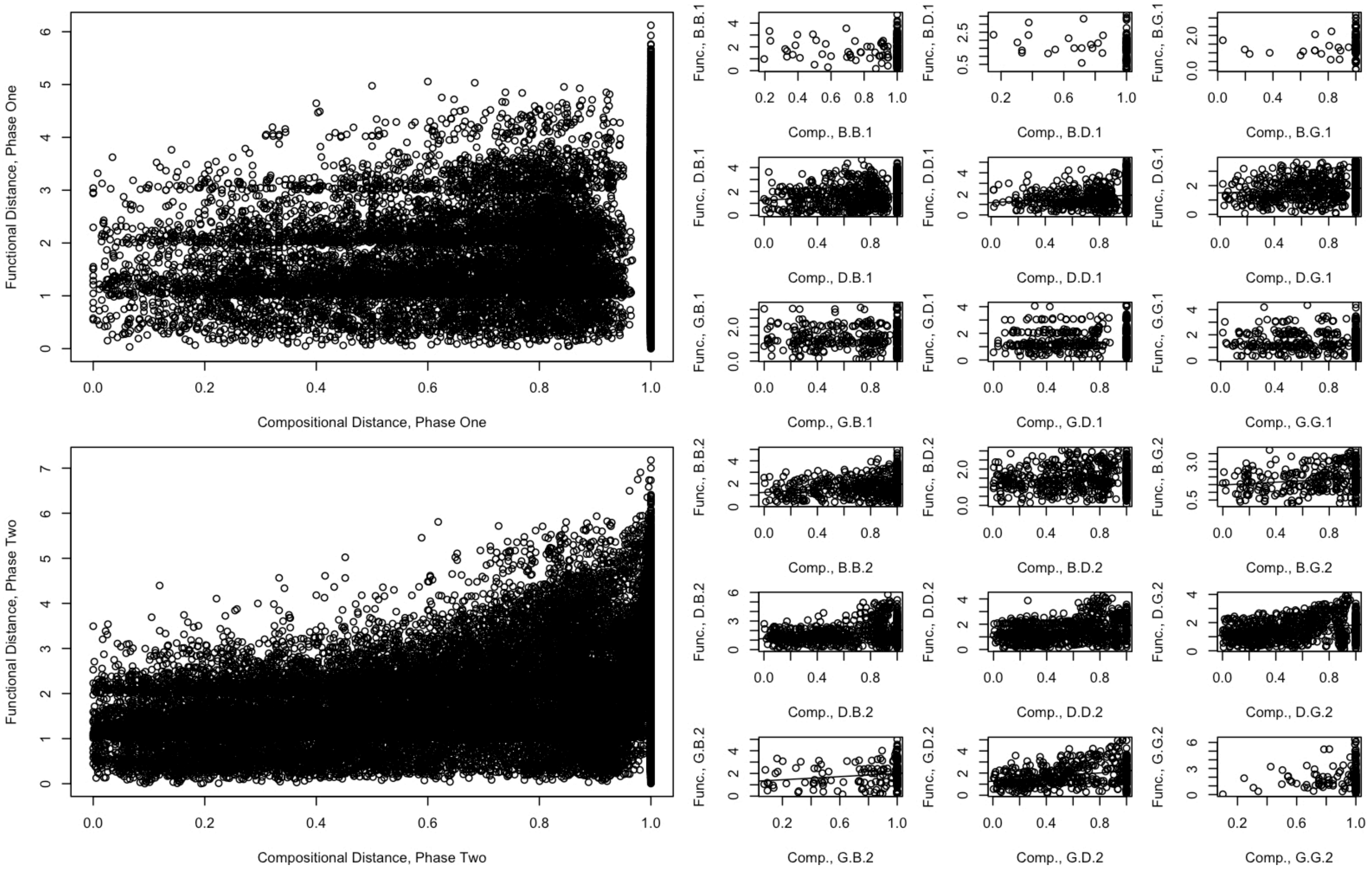

Supplemental Figure 9. Mantel tests across the study and partitioned by treatment for phase one (top 10 panels) and phase two (bottom 10 panels).

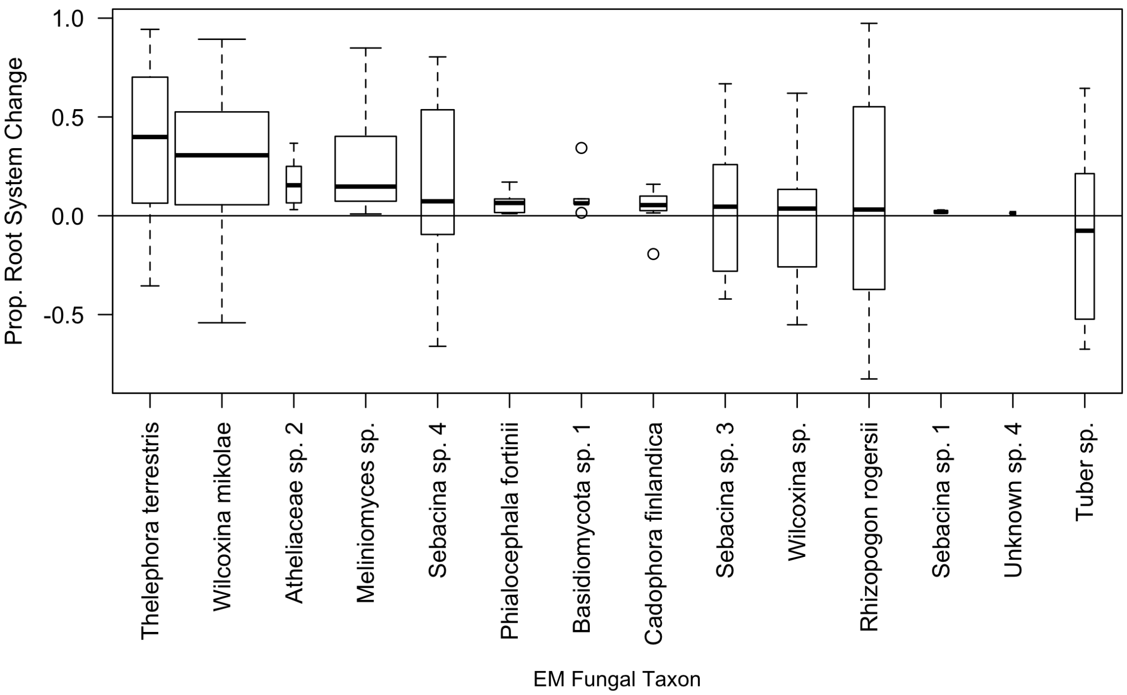

Supplemental Figure 10. Changes in fungal populations on individual seedling root systems. Boxplots show median and quartiles for changes in mycorrhization of a particular taxon on seedlings where it was present during both Phase 1 and Phase 2. Widths of boxplots are proportional to the number of seedlings on which that taxon was found.

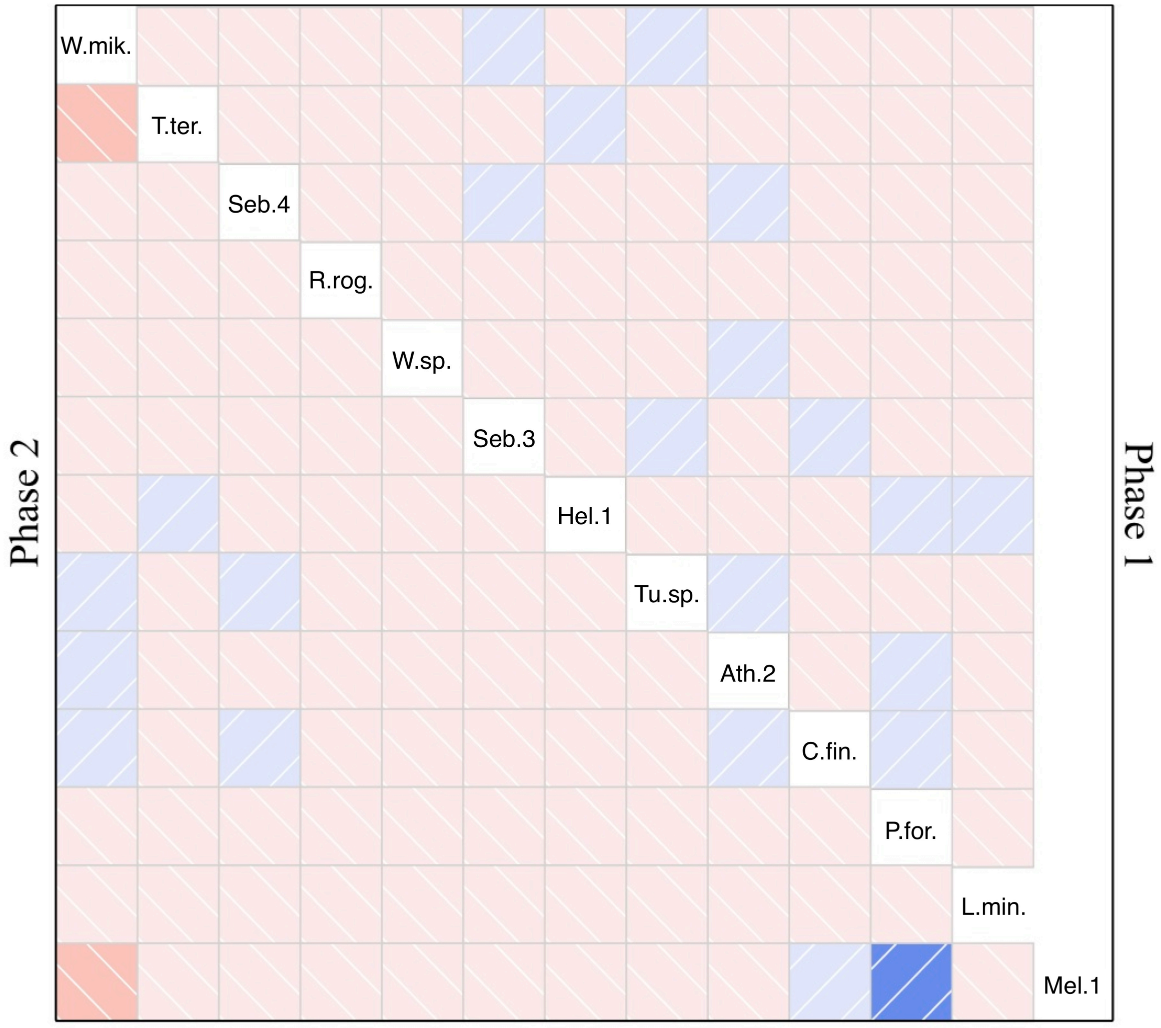

Supplemental Figure 11. Correlogram showing primarily negative relationships between fungal taxa in the study. Shades of red indicate negative relationships; blue indicates positive. Darker colors indicate significant results (*P* < 0.05). The thirteen species shown represent the intersection of the top ten most abundant taxa in Phase 1 and Phase 2 (Supplementary Figure 4).

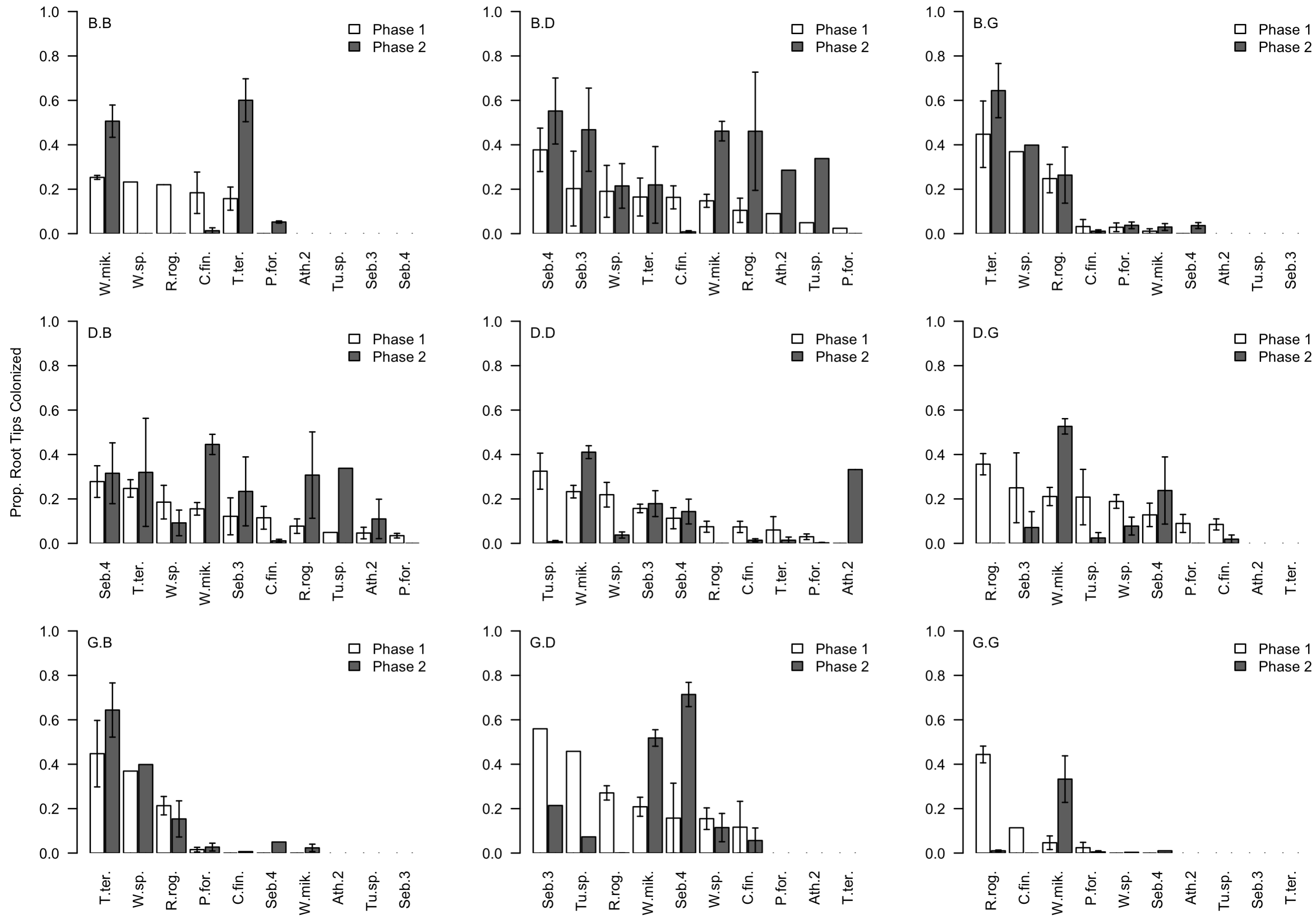

Supplementary Figure 12. Proportion of root tips in each pot occupied by each of the ten most abundant fungal taxa in the study. Data are partitioned by treatment (Self Origin . Neighbor Origin, as labeled in upper left of each panel). Bar heights are mean values with whiskers representing ± 1 standard deviation.

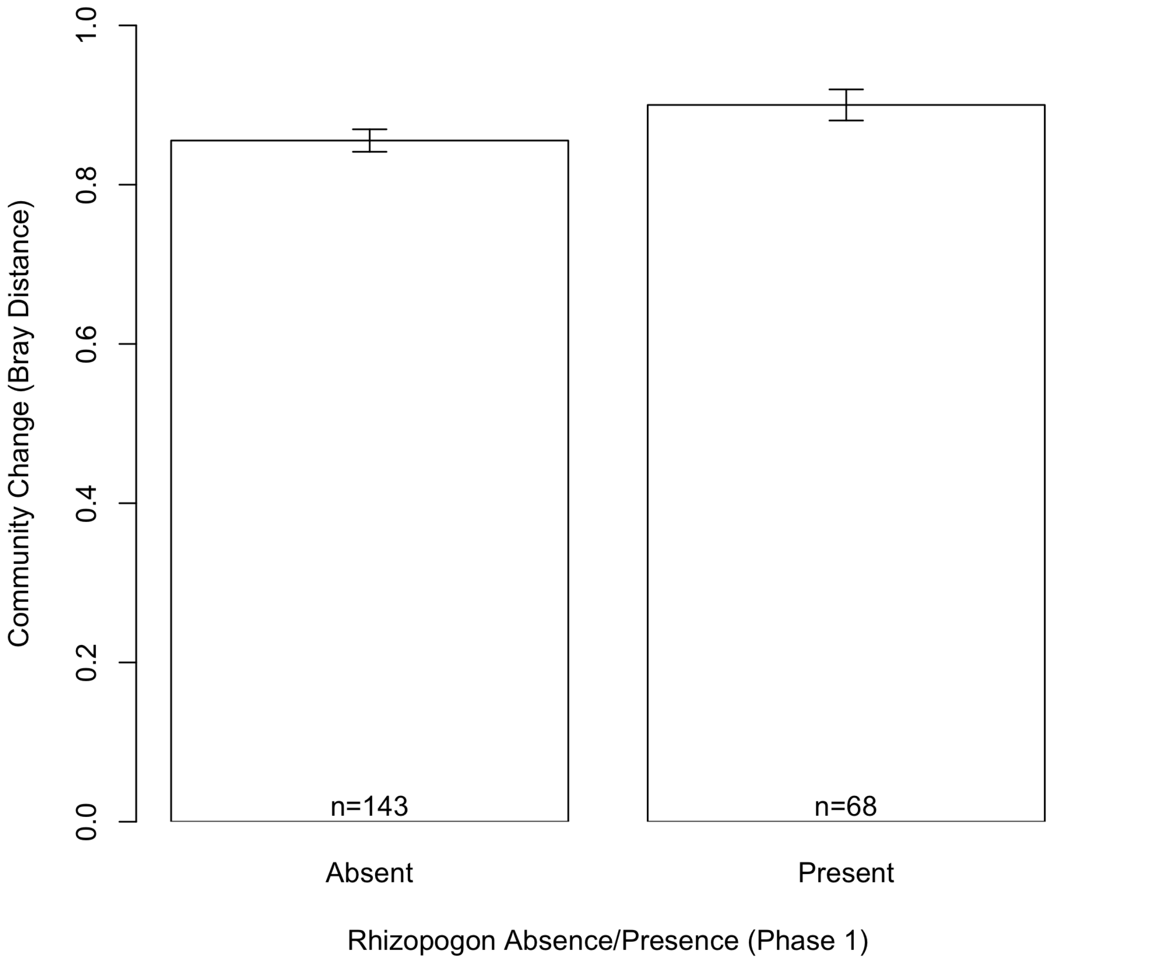

Supplemental Figure 13. *Rhizopogon rogersii* effect on change in community composition. Bar heights are mean values with whiskers representing ± 1 standard deviation. Numbers of seedlings in each group are given at the base of each bar. Where *R. rogersii* was present during Phase 1, seedling communities changed slightly more than when *R. rogersii* was absent, though this result was only marginally significant (P = 0.06, t statistic = -1.8546, df = 137.5).
